# Supplementary material for: Defining ‘therapeutic value’ of medicines: a scoping review
Source: BMJ Open. 2023 Dec 18;13(12):e078134. doi: 10.1136/bmjopen-2023-078134 (PMC10748878; doi:10.1136/bmjopen-2023-078134)
Supplement: Supplementary data [file bmjopen-2023-078134supp001.pdf]

Appendix

Search Strategy

The search string contained (value or worth) or (“value based”) or (clinical* or medical* or therap* or cur* or pay* or drug* or pharmaceutical* or evaluat* or assess* or defin*). An additional operator was used so that there was a maximum of three words between the above terms and (valu* or worth). The second search string was (clinical* or medical* or therap* or cur* or patient*) and had an adjacent operator so that there was a maximum of three words benefit. The third search string was the following: ((drug* OR pharmac* OR medicine* OR medicat*) NEAR/20 pric*) OR AB= ((drug* OR pharmac* OR medicine* OR medicat*) NEAR/3 pric)).

Search Web of Science on the 18.12.2020

1. TI=((drug* OR pharmac* OR medicine* OR medicat*) NEAR/20 pric*) OR AB=((drug* OR pharmac* OR medicine* OR medicat*) NEAR/3 pric*)
   Indexes=SCI-EXPANDED, SSCI, A&HCI, CPCI-S, CPCI-SSH, BKCI-S, BKCI-SSH, ESCI,CCR-EXPANDED, IC Timespan=All years
2. TI=(value OR worth) OR TS=("value based") OR TS=((clinical* OR medical* OR therap* OR cur* OR pay* OR drug* OR pharmaceutical* OR evaluat* OR assess* OR defin*) NEAR/3 (valu* OR worth)) OR TS=((clinical* OR medical* OR therap* OR cur* OR patient*) NEAR/3 benefit)
   Indexes=SCI-EXPANDED, SSCI, A&HCI, CPCI-S, CPCI-SSH, BKCI-S, BKCI-SSH, ESCI, CCR-EXPANDED, IC Timespan=All years
3. #2 AND #1
   Indexes=SCI-EXPANDED, SSCI, A&HCI, CPCI-S, CPCI-SSH, BKCI-S, BKCI-SSH, ESCI,
   CCR-EXPANDED, IC Timespan=All years
4. #2 AND #1
   Refined by: LANGUAGES: (ENGLISH OR FRENCH OR GERMAN OR ITALIAN ORSPANISH)
   Indexes=SCI-EXPANDED, SSCI, A&HCI, CPCI-S, CPCI-SSH, BKCI-S, BKCI-SSH, ESCI, CCR-EXPANDED, IC Timespan=All year

Search Cochrane Library on the 18.12.2020

1. ((drug* OR pharmac* OR medicine* OR medicat*) NEAR/20 pric*):ti OR ((drug* OR pharmac* OR medicine* OR medicat*) NEAR/3 pric*):ab,kw
2. (value OR worth):ti OR 'value based':ti,ab,kw OR ((clinical* OR medical* OR therap* OR cur* OR pay* OR drug* OR pharmaceutical* OR evaluat* OR assess* OR defin*) NEAR/3 (valu* OR worth)):ti,ab,kw OR ((clinical* OR medical* OR therap* OR cur* OR patient*) NEAR/3 benefit):ti,ab,kw
3. #1 AND #2
4. Type a search term or use the S or MeSH buttons to compose

Search Scopus on the 18.12.2020

1. TITLE(value OR worth) OR TITLE-ABS-KEY("value based") OR TITLE-ABS-KEY((clinical* OR medical* OR therap* OR cur* OR pay* OR drug* OR pharmaceutical* OR evaluat* OR assess* OR defin*) W/3 (valu* OR worth)) OR TITLE-ABS-KEY((clinical* OR medical* OR therap* OR cur* OR patient*) W/3 benefit)
2. TITLE ((drug* OR pharmac* OR medicine* OR medicat*) W/20 pric*) OR ABS ((drug* OR pharmac* OR medicine* OR medicat*) W/3 pric*)) AND (TITLE (value OR worth) OR TITLE-ABS-KEY ("value based") OR TITLE-ABS-KEY ((clinical* OR medical* OR therap* OR cur* OR pay* OR drug* OR

pharmaceutical* OR evaluat* OR assess* OR defin*) W/3 (valu* OR worth)) OR TITLE-ABS-KEY ((clinical* OR medical* OR therap* OR cur* OR patient*) W/3 benefit)) AND (LIMIT-TO (LANGUAGE , "English") OR LIMIT-TO (LANGUAGE , "French") OR LIMIT-TO (LANGUAGE , "German") OR LIMIT-TO (LANGUAGE , "Spanish") OR LIMIT-TO (LANGUAGE , "Italian"))

3. (TITLE ((drug* OR pharmac* OR medicine* OR medicat*) W/20 pric*) OR ABS ((drug* OR pharmac* OR medicine* OR medicat*) W/3 pric*)) AND (TITLE (value OR worth) OR TITLE-ABS-KEY ("value based") OR TITLE-ABS-KEY ((clinical* OR medical* OR therap* OR cur* OR pay* OR drug* OR pharmaceutical* OR evaluat* OR assess* OR defin*) W/3 (valu* OR worth)) OR TITLE-ABS-KEY ((clinical* OR medical* OR therap* OR cur* OR patient*) W/3 benefit))
4. TITLE (value OR worth) OR TITLE-ABS-KEY ("value based") OR TITLE-ABS-KEY ((clinical* OR medical* OR therap* OR cur* OR pay* OR drug* OR pharmaceutical* OR evaluat* OR assess* OR defin*) W/3 (valu* OR worth)) OR TITLE-ABS-KEY ((clinical* OR medical* OR therap* OR cur* OR patient*) W/3
5. TITLE ((drug* OR pharmac* OR medicine* OR medicat*) W/20 pric*) OR ABS ((drug* OR pharmac* OR medicine* OR medicat*) W/3 pric*)

Search Regional Business News on the 18.12.2020

1. TI ((drug* OR pharmac* ORmedicine* OR medicat*) N20pric*) OR AB ((drug* OR pharmac* OR medicine* OR medicat*) N3 pric*)
2. TI (value OR worth) OR TI ("value based") OR TI ((clinical* OR medical* OR therap* OR cur* OR pay* OR drug* OR pharmaceutical* OR evaluat* OR assess* OR defin*) N3 (valu* OR worth)) OR TI ((clinical* OR medical* OR therap* OR cur* OR patient*) N3 benefit) OR AB ("value based") OR AB ((clinical* OR medical* OR therap* OR cur* OR pay* OR drug* OR pharmaceutical* OR evaluat* OR assess* OR defin*) N3 (valu* OR worth)) OR AB ((clinical* OR medical* ORtherap* OR cur* OR patient*) N3 benefit)
3. S1 AND S2

Search Medline on the 18.12.2020

1. (((MH "Drug Costs") OR (MH "Pharmaceutical Preparations+")) AND TX (pric*)) OR TI ((drug* OR pharmac* OR medicine* OR medicat*) N20 pric*) OR AB ((drug* OR pharmac* OR medicine* OR medicat*) N3 pric*
2. TI (value OR worth) OR TX ("value based") OR TX ((clinical* OR medical* OR therap* OR cur* OR pay* OR drug* OR pharmaceutical* OR evaluat* OR assess* OR defin*) N3 (valu* OR worth)) OR TX((clinical* OR medical* ORtherap* OR cur* OR patient*)N3 benefit)
3. S1 AND S2
4. S1 AND S2 with Limiters - Language:English, French, German, Italian, Spanish Expanders – Apply equivalent subjects Search modes - Find all mysearch term

Search Embase Session Results on the 18.12.2020

1. ('pharmacoeconomics'/exp OR 'drug'/exp) AND 'pric*':ti,ab,kw OR (((drug* OR pharmac* OR medicine* OR medicat*) NEAR/20 pric*):ti) OR (((drug* OR pharmac* OR medicine* OR medicat*) NEAR/3 pric*):ab,kw)
2. value:ti OR worth:ti OR 'value based':ti,ab,kw OR (((clinical* OR medical* OR therap* OR cur* OR pay* ORdrug* OR pharmaceutical* OR evaluat* OR assess* OR defin*)

NEAR/3 (valu* OR worth)):ti,ab,kw) OR (((clinical* OR medical* OR therap* OR cur* OR patient*) NEAR/3 benefit):ti,ab,kw)

3. #1 AND #2
4. #1 AND #2 AND [conference abstract]/lim
5. #1 AND #2 NOT [conference abstract]/lim
6. #1 AND #2 NOT [conference abstract]/lim AND ([english]/lim OR [french]/lim OR [german]/lim OR [italian]/lim OR [spanish]/lim)

EconLit on the 18.12.2020

1. TI ((drug* OR pharmac* OR medicine* OR medicat*) N20 pric*) OR AB ((drug* OR pharmac* OR medicine* OR medicat*) N3 pric*)
2. TI (value OR worth) OR TI ("value based") OR TI ((clinical* OR medical* OR therap* OR cur* OR pay* OR drug* OR pharmaceutical* OR evaluat* OR assess* OR defin*) N3 (valu* OR worth)) OR TI ((clinical* OR medical* OR therap* OR cur* OR patient*) N3 benefit) OR AB ("value based") OR AB ((clinical* OR medical* OR therap* OR cur* OR pay* OR drug* OR pharmaceutical* OR evaluat* OR assess* OR defin*) N3 (valu* OR worth)) OR AB ((clinical* OR medical* OR therap* OR cur* OR patient*) N3 benefit)
3. S1 AND S2

CINAHL on the 18.12.2020

1. ((MH "Drugs+") AND (TI (pric*) OR AB (pric*))) OR TI ((drug* OR pharmac* OR medicine* OR medicat*) N20 pric*) OR AB ((drug* OR pharmac* OR medicine* OR medicat*) N3 pric*)
2. TI (value OR worth) OR TI ("value based") OR TI ((clinical* OR medical* OR therap* OR cur* OR pay* OR drug* OR pharmaceutical* OR evaluat* OR assess* OR defin*) N3 (valu* OR worth)) OR TI ((clinical* OR medical* OR therap* OR cur* OR patient*) N3 benefit) OR AB ("value based") OR AB ((clinical* OR medical* OR therap* OR cur* OR pay* OR drug* ORpharmaceutical* OR evaluat*OR assess* OR defin*) N3(valu* OR worth)) OR AB ((clinical* OR medical* OR therap* OR cur* OR patient*)N3 benefit)
3. S1 AND S2
4. S1 AND S2

Search on Business Source Premier on 18.12.2020

1. DE "DRUG prices" OR DE "PHARMACEUTICAL reference pricing" OR TI ((drug* OR pharmac* OR medicine* OR medicat*) N20 pric*) OR AB ((drug* OR pharmac* OR medicine* OR medicat*) N3 pric*)
2. TI (value OR worth) OR TI ("value based") OR TI ((clinical* OR medical* OR therap* OR cur* OR pay* OR drug* OR pharmaceutical* OR evaluat* OR assess* OR defin*) N3 (valu* OR worth)) OR TI ((clinical* OR medical* OR therap* OR cur* OR patient*) N3 benefit) OR AB ("value based") OR AB ((clinical* OR medical* OR therap* OR cur* OR pay* OR drug* OR pharmaceutical* OR evaluat* OR assess* OR defin*) N3 (valu* OR worth)) OR AB ((clinical* OR medical* OR therap* OR cur* OR patient*) N3 benefit)
3. S1 AND S2
