# Supplementary material for: Defining ‘therapeutic value’ of medicines: a scoping review
Source: BMJ Open. 2023 Dec 18;13(12):e078134. doi: 10.1136/bmjopen-2023-078134 (PMC10748878; doi:10.1136/bmjopen-2023-078134)
Supplement: Author's manuscript [file bmjopen-2023-078134.draft_revisions.pdf]

BMJ Open

BMJ Open is committed to open peer review. As part of this commitment we make the peer review history of every article we publish publicly available.

When an article is published we post the peer reviewers' comments and the authors' responses online. We also post the versions of the paper that were used during peer review. These are the versions that the peer review comments apply to.

The versions of the paper that follow are the versions that were submitted during the peer review process. They are not the versions of record or the final published versions. They should not be cited or distributed as the published version of this manuscript.

BMJ Open is an open access journal and the full, final, typeset and author-corrected version of record of the manuscript is available on our site with no access controls, subscription charges or pay-per-view fees (http://bmjopen.bmj.com).

If you have any questions on BMJ Open's open peer review process please email info.bmjopen@bmj.com

BMJ Open

Defining "therapeutic value" of medicines: a scoping review

Journal:	*BMJ Open*
Manuscript ID	bmjopen-2023-078134
Article Type:	Original research
Date Submitted by the Author:	29-Jul-2023

Complete List of Authors:	Glaus, Camille ; University of Zurich, Rechtswissenschaftliches Institut<br>Vokinger, Kerstin; University of Zurich, Institute of Law; Harvard Medical School, Program on Regulation, Therapeutics, and Law<br>Kloeti, Andrina; University of Zurich

Keywords:	Health policy < HEALTH SERVICES ADMINISTRATION & MANAGEMENT, MEDICAL ETHICS, Health economics < HEALTH SERVICES ADMINISTRATION & MANAGEMENT

SCHOLARONE™
Manuscripts

I, the Submitting Author has the right to grant and does grant on behalf of all authors of the Work (as defined in the below author licence), an exclusive licence and/or a non-exclusive licence for contributions from authors who are: i) UK Crown employees; ii) where BMJ has agreed a CC-BY licence shall apply, and/or iii) in accordance with the terms applicable for US Federal Government officers or employees acting as part of their official duties; on a worldwide, perpetual, irrevocable, royalty-free basis to BMJ Publishing Group Ltd ("BMJ") its licensees and where the relevant Journal is co-owned by BMJ to the co-owners of the Journal, to publish the Work in this journal and any other BMJ products and to exploit all rights, as set out in our licence.

The Submitting Author accepts and understands that any supply made under these terms is made by BMJ to the Submitting Author unless you are acting as an employee on behalf of your employer or a postgraduate student of an affiliated institution which is paying any applicable article publishing charge ("APC") for Open Access articles. Where the Submitting Author wishes to make the Work available on an Open Access basis (and intends to pay the relevant APC), the terms of reuse of such Open Access shall be governed by a Creative Commons licence – details of these licences and which Creative Commons licence will apply to this Work are set out in our licence referred to above.

Other than as permitted in any relevant BMJ Author's Self Archiving Policies, I confirm this Work has not been accepted for publication elsewhere, is not being considered for publication elsewhere and does not duplicate material already published. I confirm all authors consent to publication of this Work and authorise the granting of this licence.

For peer review only

Defining "therapeutic value" of medicines: a scoping review

Camille EG Glaus, BSc, JD[1], Andrina Kloeti, MMed[1], Kerstin N Vokinger, MD, JD, PhD[1]

Affiliations: [1] Institute of Law, University of Zurich, Zurich, Switzerland.

Corresponding author: Prof Kerstin N Vokinger, Institute of Law, University of Zurich, Ramistrasse 74, 8001 Zurich, Switzerland, email: Lst.vokinger@rwi.uzh.ch

Date: April 8, 2023

Word count: 2,563

For peer review only

Abstract

Objectives: In recent years, discussions on the importance and scope of therapeutic value of new medicines have intensified, stimulated by the increase of prices and number of medicines entering the market. This study aims to perform a scoping review identifying factors contributing to the definition of the therapeutic value of medicines.

Design: Scoping review.

Data sources: We searched the MEDLINE, CINAHL, Embase, Business Source Premier, EconLit, Regional Business News, Cochrane, Web of Science, Scope and Pool databases through December 2020 in English, German, French, Italian and Spanish.

Eligibility criteria: Studies that included determinants for the definition of therapeutic value of medicines were included.

Data extraction and synthesis: Data was extracted using the mentioned data sources mentioned. Two reviewers independently screened and analyzed the articles. Data were analyzed from April 2021 to May 2022.

Results: Of the 1883 studies screened, 51 were selected and the identified factors contributing to the definition of therapeutic value of medicines were classified in three categories: Patient perspective, public health perspective, and socio-economic perspective. More than three-quarters of the included studies were published after 2014, with the majority of the studies focusing on either cancer disorders (14/51, 27.5%) or rare diseases (11/51, 21.6%). Frequently mentioned determinants for value were quality of life, therapeutic alternatives and side effects (all patient perspective), prevalence/incidence and clinical endpoints (all public health perspective), and costs (socio-economic perspective).

Conclusions: Multiple determinants have been developed to define the therapeutic value of medicines, most of them focusing on cancer disorders and rare diseases. Considering the relevance of value of medicines to guide patients and physicians in decision-making as well as policymakers in resource allocation decisions, a development of evidence-based factors for the definition of therapeutic value of medicines is needed across all therapeutic areas.

For peer review only

Strengths and limitations of this study

- This scoping review was based on a comprehensive search and articles were screened and analyzed by two independent authors.
- Potential studies could be missed due to the heterogeneity of terms.
- Another categorization of value determinants is possible.

Introduction

In recent years, discussions on the importance of value of new medicines have intensified, stimulated by the increase of prices and number of medicines entering the market.[1] Several countries with price regulations (for example Germany or France) have tasked health technology agencies to assess the added therapeutic value of new medicines as a basis for price negotiation.[2] Also medical associations developed frameworks that enable the value assessment of medicines with the goal to support physicians and patients in their decision-making. For example, the European Society for Medical Oncology (ESMO) created the ESMO-magnitude of clinical benefit scale, and the American Society of Clinical Oncology (ASCO) implemented the ASCO-value framework specifically for cancer medicines.[3,4] Additionally, non-profit organizations such as the Institute for Clinical and Economic Review (ICER) emerged with the goal of conducting and disseminating comparative effectiveness evaluations to, among other things, encourage fair pricing.[5]

Studies indicate that only a fraction of new medicines provide high added therapeutic value when applying the therapeutic rating of health technology agencies or medical associations.[6,7,8] However, value of medicines can vary depending on the underlying understanding of value.[9,10]

The importance of the determinants contributing to the assessment of therapeutic value of medicines will further increase in the upcoming years given the growth of high costs of medicines that threaten healthcare budgets across countries. To support ongoing discussions on how to define value, we undertook a scoping review of the literature to identify the determinants used to assess the therapeutic value of medicines.

Methods

Selection criteria

This scoping review was conducted using the PRISMA guidelines for scoping reviews. In December 2020, the databases Medline (PubMed), CINAHL, EMBASE, Business Source Premier, EconLit, Regional Business News, Cochrane, Web of Science, Scope and

For peer review only - http://bmjopen.bmj.com/site/about/guidelines.xhtml

Pool were searched for studies published until December 2020 in English, German, French, Italian and Spanish.

Search strategy

The search string contained (value or worth) or ("value based") or (clinical* or medical* or therap* or cur* or pay* or drug* or pharmaceutical* or evaluat* or assess* or defin*). An additional operator was used so that there was a maximum of three words between the above terms and (valu* or worth). The second search string was (clinical* or medical* or therap* or cur* or patient*) and had an adjacent operator so that there was a maximum of three words benefit. The third search string was the following: ((drug* OR pharmac* OR medicine* OR medicat*) NEAR/20 pric*) OR AB= ((drug* OR pharmac* OR medicine* OR medicat*) NEAR/3 pric)).

Screening protocol

Two authors (CEGG, AK) independently screened the articles following a fixed protocol. They both screened all abstracts and titles and titles based on the inclusion criteria. They then compared their results and established a list with the non-congruent results. Disagreement was resolved in discussion with the last author (KNV). The same procedure was followed for the screening of the full texts (**Table 1**).

Study selection

For studies that appeared from their title or abstract to discuss determinants of the therapeutic value of medicines, full articles related to these titles were obtained and screened. Studies were included if they proposed a definition and/or one or more determinants of therapeutic value of medicines. We excluded studies that provided information about the value of specific medicines but did not include specific information on determinants of value. We also excluded studies that focused on related topics but did not directly address the determinants of therapeutic value, such as value-based pricing.

Analysis

The first author (CEGG) reassessed the identified results and devided them in three categories: patient perspective, public health perspective and socio-economic perspective. This decision was discussed with the last author (KNV). Studies that matched more than one category were included separately in each category. The studies were assessed from April 2021 to May 2022. This analysis was descriptive neither the veracity nor the justifiability of the results were assessed.

The study did not require institutional review board approval because the data were based on publicly available information. The study adheres with the EQUATOR Reporting Guidelines. Detailed methods are presented in the Supplement.

For peer review only - http://bmjopen.bmj.com/site/about/guidelines.xhtml

Patient and public involvement

None.

Results

The search strategy generated 1833 records, of which we identified 442 for full review. 44 unique papers were included. Reference mining led us to 7 additional articles that met our criteria, resulting in a total of 51 articles for the final sample. **Figure 1** provides a flow diagram of the search results.

The dates of the articles range between 1996 and 2020, although fewer than one-quarter (11/50, 22%) were published prior 2014.

Of the 51 studies, most determinants of value focused on cancer medicines (14/51, 27.5%) or medicines targeting orphan diseases (11/51, 21.6%). 5/51 (9.8%) were for other therapeutic areas (such as chronic diseases[11] or neurologic diseases[12]) and 21/51 (41.2%) were unspecified.

Some studies outlined a holistic approach for the assessment of value of medicines. For example, Schnipper and colleagues outlined that value is generally accepted as a measure of outcomes achieved per monetary expenditure.[3] However, in most cases the studies did not incorporate a holistic approach, but rather focused on a perspective (e.g., patient perspective) and on specific determinants (**Tables 2 – 4**). We divided the different determinants of value in three categories. Some determinants can be classified in more than one category. For example, we classified the determinant 'effectiveness' under the public policy perspective, alternatively it could also fall into the patient perspective.

Value of medicines: Patient perspective

We identified 48 articles that matched this category, focusing on the following aspects: Quality of life (31 studies)[3,4,11–39], burden of disease (including severity of the disease, unmet medical need, 39 studies)[3,11,13,15–17,19–22,24–32,34–37,39–54] and convenience (e.g., administration route, 16 studies)[3,11–13,15,22,24,28,29,31,35,36,49,52,53,55] (**Table 2**).

Quality of life

Many studies acknowledged the importance of quality of life when assessing the value of medicines.[28,36] However, most studies did not offer a clear or unified definition for this determinant. Some studies specified quality of life as the impact of the treatment on the physical and mental abilities.[3,30,31,35] Another study highlighted that quality of life for cancer patients should be assessed in a questionnaire that addresses four categories: social well-

For peer review only - http://bmjopen.bmj.com/site/about/guidelines.xhtml

being, emotional well-being, memory and need of a caregiver.[27] And a further study emphasized that quality of life is inherent to the characteristics of the individual patient.[11]

Burden of disease

A unified definition could also not be identified for the value determinant burden of disease. For example, different definitions were applied for the determinant "severity". One study stated that "severity of the disease relates to the condition's degree of seriousness in response to mortality and morbidity-derived disability […] or the expected remaining life years adjusted for their quality of life.[56] Another study defined severity more broadly as the "overall impact of a problem on an individual."[25] This was specified in other studies, which stated that the severity of a disease included physical and mental aspects of the disease.[25,26,35]

Unmet medical need was a further frequently mentioned determinant for the evaluation of value of medicines, especially in the context of orphan diseases.[37,47,46,35,43,26] Unmet medical need was often defined as the therapeutic options for the patient or the number of available medicines for the specific disease.[21,26,35,41,42,47] Some studies underlined that the benefits of alternative medicines should also be considered.[35,47] Medicines targeting a disease with no alternative treatment option available should be granted a higher value.[47]

<u>Value of medicines: Public health perspective</u>

49 studies in our study cohort focused on this perspective when assessing value of medicines. We identified the following clusters: Epidemiological endpoints (e.g., size of population, 13 studies)[14,15,24,26,35,37,40,42,45,46,50,57,58] and clinical endpoints (e.g., safety, efficacy or evidence, 45 studies)[3,4,12,13,15–25,27–33,35–37,39–42,44–55,57–60] (**Table 3**).

Epidemiological endpoints

Multiple studies referred to epidemiological endpoints (for example, incidence or prevalence), as an important aspect for the evaluation of value of medicine (10 studies).[15,24,26,35,37,42,45,46,50,57] Some studies argued that the rarity of a disease increases the value of the medicines targeting those diseases,[25,26,35] while others stated that this approach is unethical.[24,58] Some studies also argued that taking into account epidemiological endpoints would be unethical since that would unjustifiably increase the treatment value of certain diseases compared to others.[24,58]

Clinical endpoints

Many studies considered outcomes of clinical trials, such as safety or effectiveness as core determinants for the evaluation of value of medicines.[3,4,12–20,22–33,35,37,39–

For peer review only - http://bmjopen.bmj.com/site/about/guidelines.xhtml

[42,44–55,58–60], even more so in more recent studies.[16,22,24,32] Trial outcomes are influenced by the understanding of the disease,[37] the stage of the disease (the benefit of medicines targeting the treatment of advanced-stage diseases may be more modest compared to those applied in a curative setting),[3,16,31,38] the duration of the study (some medicines result in a short tumor response and in case of a short study duration the long-term effects may be unclear),[16] the size of the targeted population evaluated,[26,37,47,50] the study design,[24,26] the subjects of the study and the potential resulting heterogeneity in medicine response.[11,16,22,27,32,60] Concerns with regard to the lack of evidence was particularly raised for medicines targeting orphan diseases and cancer medicines.[26,37,47,50] Value of medicines based on outcomes of clinical trials may change over a medicine's lifecycle depending on, among other things, new evidence provided in further trials,[16,32,58] additional indications that increase the aggregated value to society,[13,24,59] and outcomes in long-term studies.[16]

Side effects and toxicity were other important determinants frequently highlighted as determinants for the assessment of value of medicines.[3,4,15,16,21–25,27,28,30,31,33,36,37,39,48,50,59] Multiple studies specified side effects and toxicity as tolerability, discontinuation or complications.[12,13,21,24,35] In the same context, several authors stressed that compliance to a medicine is an important element of value.[13,20,22,25,28–30,32,33,35,42,53,54]

<u>Value of medicines: Socio-economic perspective</u>

We included 46 studies in the study cohort. We identified the following clusters: Economic burden (e.g., treatment costs, non-treatment costs, and willingness to pay, 21 studies)[3,11–16,22,24,25,28,29,31–34,36,44,49,51,60], innovation (e.g., mechanism of action or spill-over effect, latter includes, for example, benefits that may evolve due to the treatment of the disease, 26 studies)[11,13,17,19,22,24–26,32,34–36,40–45,49,51–55,59,60], and broader social impact (e.g., equity, 23 studies)[3,15–17,24,27,28,31,33,34,36–38,42,45,49,50,53–55,58–60] (**Table 4**).

Economic burden

Treatment costs and non-treatment costs (e.g., caregiver costs, lost work productivity) were considered by multiple studies as important determinants to appraise value of medicines.[3,11–16,22,24,25,29,31–34,36,42,44,49,60] Non-treatment costs include also the financial burden for society, which was identified as a relevant determinant for the assessment of value of medicines.[11,24,33,37,38,49,50]

Some studies defined value as the willingness to pay for a medicine.[11,14,15,28,30,32,33,51] One study underlined the subjectivity of this criteria, highlighting that, for example, patients may have another willingness compared to physicians and that the willingness to pay can also vary among patients or physicians.[11]

For peer review only

Innovation

Many studies argued that innovation is another core element when assessing value of medicines.[11,13,17,19,22,24–26,32,34–36,40–45,49,51–55,59,60] There was not one unified definition for innovation. Examples included the medicine's novel mechanism of action,[13,19,24,42,51,52,54,55] its effectiveness and side effects,[48] its spill-over effect (understood as the gained knowledge during the development of the medicine that serves as a basis for the development of further medicines),[13,24,34,36,41,45,59,60] or the route of medicine administration.[13]

Other identified determinants for the evaluation of value of medicines were the research and development costs,[26,33,35] or the level of complexity of the medicine.[26,35,43] Another study explicitly stated that research and development costs should not be considered for the assessment of value, arguing that value should only reflect the direct benefits for the patient.[14]

Social impact

Some studies considered the broader social impact of medicines as important elements for the assessment of value. It includes general ethical principles, such as altruism towards the poor,[59] discrimination,[17,49] fairness,[40] or equity.[3,31,34,37,38,42,48,58] It also comprises other societal determinants such as prevention,[24,25,38] and the relief of family members of emotionally and physically demanding responsibilities.[25,27,28,37,59]

Discussion

More than three quarter of the included studies were published after 2014, with the majority of the studies focusing on either cancers or rare diseases. A major amount of the included studies highlighted only specific aspects of value of medicines that we classified in three categories: Patient perspective, public health perspective, and socio-economic perspective.

Our study findings have implications for the ongoing discussions around value of medicines. Value assessment of medicines support, for example, patients and physicians in decision-making in selecting the best treatment for an individual patient, or health technology agencies and policymakers in resource allocation and reimbursement decisions.[3,61] Given the importance of value assessment of medicines, our study findings suggest that more analysis and discussions are indicated to develop an evidence-based definition for and understanding of value. The identified determinants in this study for the value of medicines could serve as a

For peer review only - http://bmjopen.bmj.com/site/about/guidelines.xhtml

basis in this regard. Frequently mentioned determinants for value were quality of life, therapeutic alternatives and side effects (all patient perspective), prevalence/incidence and clinical endpoints (all public health perspective), and costs (socio-economic perspective). The specific determinants for the value assessment of medicines may vary depending on the country, health care system, therapeutic area and patient population.[3]

Value determinants, such as a medicine's evidence, clinical outcomes or its impact on quality of life can change over time based either on new findings from additional clinical trials or real-world evidence or in comparison to new medicines entering the market for the same indication.[59] These study findings suggest that the value of medicines should not be understood statically but rather dynamically, which is consistent with the approach of HTA bodies or agencies that re-assess value and prices of medicines either on a regular basis (e.g., every three years in Switzerland) or if new evidence from clinical trials or real-world data is provided (e.g., Germany).

Many of the studies in the study cohort were published in recent years, with the majority of the studies focusing on either cancers (14/51, 27.5%) or rare diseases (11/51, 21.6%). The focus on these medicines might be explained by their high treatment prices, their potentially severe adverse events and the increasing amount of medicines in these therapeutic areas entering the market.[62,63] Furthermore, medical associations such as ASCO and ESMO developed value frameworks specifically for cancer medicines. By contrast, for example, only one included study focused on the determinants of value specifically for diabetic medicines.[18] Also other therapeutic areas, such as psychiatry or neurology, are not or only marginally addressed in the included studies. A stronger focus on the factors of value of medicines in general and for therapeutic areas other than cancer and rare diseases is indicated.

<u>Limitations</u>

This study has limitations. Due to the broadness of the topic, it is possible that the study cohort is not exhaustive. Our multilayered search strategy allowed us to screen a wide range of studies, leaving us confident that we reached thematic saturation for main outcomes. However, we focused only on determinants defining the therapeutic value of medicines and excluded the rich literature on related topics such as "value in healthcare" or "health care services." Furthermore, we only focused on studies published in English, German, French, Italian or Spanish. Thus, it is possible that we did not include relevant studies published in other languages. Lastly, another categorization of value determinants is possible.

For peer review only - http://bmjopen.bmj.com/site/about/guidelines.xhtml

Conclusions

Multiple determinants have been developed to define the therapeutic value of medicines. Most definitions and determinants identified for the assessment of therapeutic value of medicines were developed for cancer disorders and rare diseases. Considering the relevance of value of medicines to guide patients and physicians in decision-making as well as policymakers in resource allocation decisions, a development of evidence-based factors for the definition of therapeutic value of medicines is needed, addressing also diseases outside cancer disorders and orphan diseases.

For peer review only

Figure 1. Flow diagram of search results.

Table 1. Criteria for study selection

Inclusion	Exclusion
• Terms "value", "benefit" if determinants of value of medicine or as argument for pricing<br>• New tool/concept or proposed adoption of already existing tool/concept<br>• Tool/concepts by countries/state institutions if these countries/state institutions developed/adapted them or if they are unclear<br>• Journals<br>• Conference papers	• No direct link to "value", "HTA", "benefit" (e.g. accessibility, innovation)<br>• Application of already existing toolsconcepts<br>• Tool/concept by countries/state institutions if they only apply an already existing tool/concept<br>• Complementary medicines<br>• Biosimilars<br>• Generics<br>• Drugs (e.g. randomized clinical trials)<br>• Contracting and insurance design<br>• News articles

Table 2. Identified determinants for the assessment of value of medicines from the patient perspective

**Patient perspective**				
**Category**	**Medical speciality**			
	**Oncology (N = 14)**	**Orphan disease (N = 11)**	**Other (N = 5)**	**Not specified (N = 21)**
**Quality of life**	3, 4, 13, 15, 16, 21, 23, 27, 29, 31	17, 26, 35, 37	11, 12, 18, 30	14, 19, 20, 22, 24, 25, 28, 32, 33, 34, 36, 38, 39
**Burden of disease**				
• General definition	29, 41, 42	17, 37, 43, 44, 46, 47	30	19, 24, 32, 36, 49, 54

For peer review only - http://bmjopen.bmj.com/site/about/guidelines.xhtml

• Severity of disease, symptoms	3, 13, 15, 16, 21, 27, 31, 40, 41	17, 26, 35, 37, 43–47, 50	11, 30, 48	24, 25, 28, 32, 34, 36, 39, 49, 51, 52, 54
• Unmet medical need, alternatives	15, 21, 29, 40–42	26, 35, 37, 43, 46, 47	30	19, 20, 22, 24, 28, 32, 49, 52–54
• Others: patient experience, fear of contagion, daily activity, reaching a landmark life event, dignity	3, 16, 21, 27, 29, 31, 42	37	11	19, 20, 25, 36, 38, 49, 53
**Convenience**				
• Posology, route of administration, instructions, comfort	3, 13, 15, 29, 31	35	11, 12	22, 24, 28, 36, 49, 52, 53, 55

Note: The numbers listed in the medical specialty match the references. Studies that address multiple categories for the definition of value of medicines were listed for each category separately.

Table 3. Identified determinants for the assessment of value of medicines from the public health perspective

**Public health perspective**				
**Category**	**Medical specialty**			
	**Oncology (N = 14)**	**Orphan disease (N = 11)**	**Other (N = 5)**	**Not specified (N = 21)**
**Epidemiological endpoints**				
• Prevalence, incidence, rarity	15, 40, 42, 57	26, 35, 37, 45, 46, 50		24
**Clinical endpoints and evidence**				
• Safety	3, 16, 29, 31	35, 44	12, 48	19, 20, 22, 24, 28, 32, 33, 53, 55
• Efficacy	13, 15, 16, 29	35, 44, 50		19, 20, 22, 24, 32, 33, 52-55
• Effectiveness	3, 13, 15, 31, 40, 42	26, 35, 37	18, 30, 48	14, 20, 25, 33, 51, 53, 59, 60
• Clinical benefits	3, 4, 16, 23, 27, 29, 31, 41	17, 26, 35, 45–47, 58	30	28, 39, 49, 51, 54, 59
• Side effects, toxicity	3, 4, 13, 15, 16, 21, 23, 27, 31	37	48, 30	22, 24, 25, 28, 33, 36, 39
• Compliance, discontinuation, tolerability	13, 29	35	30	20, 22, 25, 28, 32, 33, 42, 53, 54
• Certainty, evidence	3, 16, 31, 42, 57	26, 37, 47, 50, 58		19, 22, 24, 25, 32, 36, 39, 51, 52, 60

Note: The numbers listed in the medical specialty match the references. Studies that address multiple categories for the definition of value of medicines were listed for each category separately.

Table 4. Identified determinants for the assessment of value of medicines from the socioeconomic perspective.

Socio-economic perspective				
**Category**	**Medical specialty**			
	**Oncology (N = 14)**	**Orphan disease (N = 11)**	**Other (N = 5)**	**Not specified (N = 21)**
**Economic burden**				
• Treatment costs	3, 13, 29, 31	44	11, 12	22, 24, 33, 34, 36
• Non-treatment costs	3, 15, 16, 29, 31, 42	44	11	14, 22, 24, 25, 32, 34, 36, 49, 60
• Willingness to pay	15		11, 30	14, 28, 32, 33, 51
**Innovation**				
• Innovation	40, 41	17, 44	11	22, 24, 25, 32, 36, 49, 52, 53
• Spill-over in research	13, 41	45		24, 34, 36, 49, 59, 60
• Mechanism of action, effectiveness, route of administration	13, 42		48	19, 24, 51, 52, 54, 55
• Others: research undertaken, unique indication, complexity of innovation, techological considerations	41	26, 35, 43		33, 54, 59
**Broader social impact**				
• Social value				28, 33, 36, 49, 59
• Altruism				59
• Discrimination		17		49
• Equity, fairness	3, 31, 40, 42	37, 58	48	34, 38
• Family benefit	27	37		25, 28, 59
• Others: prevention, risk reduction, feasability (adoption), public health benefits	15, 16	45, 50		24, 25, 38, 53, 54, 60

For peer review only

Note: The numbers listed in the medical specialty match the references. Studies that address multiple categories for the definition of value of medicines were listed for each category separately.

Funding: This study was funded by the Swiss National Science Foundation (SNSF, grant number 194607).

Competing interests: The authors declare no competing interests.

Data availability statement: All the data included in the study are publicly available. Data are available upon request.

Contributorship statement: CEEG contributed to the data collection, data analysis, data interpretation, first rudimentary draft, and revision of the manuscript. AK contributed to the data collection and data analysis. KNV contributed to the acquisition of funding, conception, data collection, data interpretation, first draft, and revision of the manuscript. All authors have access to all the data in the manuscript.

Ethics approval statement: The study did not require institutional review board approval because the data were based on publicly available information and no patients were involved.

For peer review only

References

1. Darrow, J. J., Avorn, J. & Kesselheim, A. S. FDA Approval and Regulation of Pharmaceuticals, 1983-2018. *JAMA* **323**, 164 (2020).
2. Vokinger, K. N. *et al.* Prices and clinical benefit of cancer drugs in the USA and Europe: a cost-benefit analysis. *The Lancet. Oncology* **21**, 664–670 (2020).
3. Schnipper, L. E. *et al.* American Society of Clinical Oncology Statement: A Conceptual Framework to Assess the Value of Cancer Treatment Options. *JCO* **33**, 2563–2577 (2015).
4. Cherny, N. I. *et al.* A standardised, generic, validated approach to stratify the magnitude of clinical benefit that can be anticipated from anti-cancer therapies: the European Society for Medical Oncology Magnitude of Clinical Benefit Scale (ESMO-MCBS). *Annals of Oncology* **26**, 1547–1573 (2015).
5. ICER. Institute For Clinical And Economic Review.
6. Hwang, T. J., Ross, J. S., Vokinger, K. N. & Kesselheim, A. S. Association between FDA and EMA expedited approval programs and therapeutic value of new medicines: retrospective cohort study. *BMJ* m3434 (2020) doi:10.1136/bmj.m3434.
7. Hwang, T. J., Vokinger, K. N. & Kesselheim, A. S. New Treatments for Migraine—Therapeutic Ratings and Comparative Coverage in the US, Canada, and Europe. *JAMA Intern Med* **182**, 101 (2022).
8. Vokinger, K. N., Hwang, T. J., Glaus, C. E. G. & Kesselheim, A. S. Therapeutic Value Assessments of Novel Medicines in the US and Europe, 2018-2019. *JAMA Netw Open* **5**, e226479 (2022).
9. Hu, I., Zheng, J. & Hu, X. H. Comparison Of Health Technology Assessment (Hta) Rankings By German And French Hta Agencies. *Value in Health* **18**, A96–A97 (2015).
10. Cherny, N. I. *et al.* Comparative Assessment of Clinical Benefit Using the ESMO-Magnitude of Clinical Benefit Scale Version 1.1 and the ASCO Value Framework Net Health Benefit Score. *JCO* **37**, 336–349 (2019).

For peer review only

11. Koh, C. Y. C. & Seager, T. P. Value-Based Pharmaceutical Pricing From the Patient Perspective Could Incentivize Innovation. *Pharmaceutical Medicine* **31**, 149–153 (2017).

12. Crowell, J. L. & Burns, T. M. Rising Drug Costs for Neurologic Diseases. *Continuum (Minneapolis, Minn.)* **26**, 1392–1406 (2020).

13. Angelis, A. *et al.* Multiple Criteria Decision Analysis for HTA across four EU Member States: Piloting the Advance Value Framework. *Social Science and Medicine* **246**, (2020).

14. Cohen, J. T., Anderson, J. E. & Neumann, P. J. Three Sets of Case Studies Suggest Logic and Consistency Challenges with Value Frameworks. *Value in Health* **20**, 193–199 (2017).

15. Doyle, J. J., Hawryluk, E., Niemira, J. & Wood, B. Evidence-Based Valuation in Oncology:: Lessons Learned from a Case Study. *Therapeutic innovation & regulatory science* **53**, 403–411 (2019).

16. Jönsson, B. & Wilking, N. New cancer drugs in Sweden: Assessment, implementation and access. *Journal of Cancer Policy* **2**, 45–62 (2014).

17. Kiernan, F. The future of pharmacoeconomic policy - does value-based pricing really have a role? *Journal of Pharmaceutical Health Services Research* **7**, 5–9 (2016).

18. Cheng, W. H., Gaudette, É. & Goldman, D. P. PCSK9 Inhibitors Show Value for Patients and the US Health Care System. *Value in Health* **20**, 1270–1278 (2017).

19. Abedi, S. The Payer Perspective. *Pharmaceutical Executive Global Digest* **35**, 40–41 (2015).

20. DeKoven, M., Hazard, E. H., Goldberg, E. & Pokras, S. Got value? Determine it. Demonstrate it. Communicate it. Realise it. *Journal of Commercial Biotechnology* **14**, 299–306 (2008).

For peer review only

21. Fonseca, R. & Hinkel, J. Value and Cost of Myeloma Therapy-We Can Afford It. *American Society of Clinical Oncology educational book. American Society of Clinical Oncology. Annual Meeting* **38**, 647–655 (2018).

22. Gregson, N., Sparrowhawk, K., Mauskopf, J. & Paul, J. Pricing medicines: theory and practice, challenges and opportunities. *Nature reviews. Drug discovery* **4**, 121–130 (2005).

23. Cherny, N. I. *et al.* ESMO-Magnitude of Clinical Benefit Scale version 1.1. *Annals of Oncology* **28**, 2340–2366 (2017).

24. Angelis, A. & Kanavos, P. Multiple Criteria Decision Analysis (MCDA) for evaluating new medicines in Health Technology Assessment and beyond: The Advance Value Framework. *Social science & medicine (1982)* **188**, 137–156 (2017).

25. Dionne, F., Mitton, C., Dempster, B. & Lynd, L. D. Developing a multi-criteria approach for drug reimbursement decision making: an initial step forward. *Journal of population therapeutics and clinical pharmacology = Journal de la therapeutique des populations et de la pharmacologie clinique* **22**, e68–e77 (2015).

26. Hughes-Wilson, W., Palma, A., Schuurman, A. & Simoens, S. Paying for the Orphan Drug System: break or bend? Is it time for a new evaluation system for payers in Europe to take account of new rare disease treatments? *Orphanet journal of rare diseases* **7**, 74 (2012).

27. Lorgelly, P. *et al.* Outcome-Based Payment Schemes: What Outcomes Do Patients with Cancer Value? *The patient* **13**, 599–610 (2020).

28. Puig-Junoy, J. & Peiró, S. From the therapeutic utility to the added therapeutic value and the incremental cost-effectiveness ratio. *Revista Espanola de Salud Publica* **83**, 59–70 (2009).

29. Rothenberg, M. L., McCarthy, J. & Holmes, P. Price, Cost, and Value of Cancer Medicines: A Pharmaceutical Industry Perspective. *Cancer journal (Sudbury, Mass.)* **26**, 281–286 (2020).

For peer review only - http://bmjopen.bmj.com/site/about/guidelines.xhtml

30. Tugwell, P. Economic evaluation of the management of pain in osteoarthritis. *Drugs* **52 Suppl 3**, 48–58 (1996).

31. Schnipper, L. E. *et al.* Updating the American Society of Clinical Oncology Value Framework: Revisions and Reflections in Response to Comments Received. *JCO* **34**, 2925–2934 (2016).

32. Adami, S. *et al.* Therapeutic value and innovation of a drug and criteria for evaluation: A multidisciplinary experience in the veneto region. *Global and Regional Health Technology Assessment* **2**, 107–113 (2015).

33. Capri, S. & Levaggi, R. Pricing policies in the pharmaceutical sector. *Drug Information Journal* **36**, 453–464 (2002).

34. Mejía, A., Gilardino, R., Kristensen, F. B. & Garrison, L. P. Value-Based Pricing in Latin America: How Far Away Are We? *Value in Health Regional Issues* **17**, 219–223 (2018).

35. Schey, C., Krabbe, P. F. M., Postma, M. J. & Connolly, M. P. Multi-criteria decision analysis (MCDA): Testing a proposed MCDA framework for orphan drugs. *Orphanet Journal of Rare Diseases* **12**, (2017).

36. Towse, A. & Barnsley, P. Approaches to identifying, measuring, and aggregating elements of value. *International journal of technology assessment in health care* **29**, 360–364 (2013).

37. Annemans, L. *et al.* Recommendations from the European Working Group for Value Assessment and Funding Processes in Rare Diseases (ORPH-VAL). *Orphanet journal of rare diseases* **12**, 50 (2017).

38. Danzon, P. M. Affordability Challenges to Value-Based Pricing: Mass Diseases, Orphan Diseases, and Cures. *Value in health : the journal of the International Society for Pharmacoeconomics and Outcomes Research* **21**, 252–257 (2018).

For peer review only

39. Skipka, G. *et al.* Methodological approach to determine minor, considerable, and major treatment effects in the early benefit assessment of new drugs. *Biometrical journal. Biometrische Zeitschrift* **58**, 43–58 (2016).

40. Linley, W. G. & Hughes, D. A. Societal views on NICE, cancer drugs fund and value-based pricing criteria for prioritising medicines: a cross-sectional survey of 4118 adults in Great Britain. *Health economics* **22**, 948–964 (2013).

41. Parmar, A., Jiao, T., Saluja, R. & Chan, K. K. W. Value-based pricing: Toward achieving a balance between individual and population gains in health benefits. *Cancer medicine* **9**, 94–103 (2020).

42. Augustovski, F. & McClellan, M. B. Current Policy and Practice for Value-Based Pricing. *Value in Health* **22**, S4–S6 (2019).

43. Picavet, E., Cassiman, D. & Simoens, S. Reimbursement of orphan drugs in Belgium: What (else) matters? *Orphanet Journal of Rare Diseases* **9**, (2014).

44. Nuijten, M. & Capri, S. Pricing of orphan drugs in oncology and rare diseases. *Journal of market access & health policy* **8**, 1838191 (2020).

45. Horgan, D. Treatment Is Worth a Lot - But Just How Much. *Biomedicine hub* **2**, 202–211 (2017).

46. Medic, G. *et al.* Do payers value rarity? An analysis of the relationship between disease rarity and orphan drug prices in Europe. *Journal of market access & health policy* **5**, 1299665 (2017).

47. Gutierrez, L., Patris, J., Hutchings, A. & Cowell, W. Principles for consistent value assessment and sustainable funding of orphan drugs in Europe. *Orphanet journal of rare diseases* **10**, 53 (2015).

48. Huskamp, H. A. Prices, profits, and innovation: examining criticisms of new psychotropic drugs' value. *Health affairs (Project Hope)* **25**, 635–646 (2006).

For peer review only - http://bmjopen.bmj.com/site/about/guidelines.xhtml

49. Sussex, J., Towse, A. & Devlin, N. Operationalizing value-based pricing of medicines : a taxonomy of approaches. *PharmacoEconomics* **31**, 1–10 (2013).

50. Denis, A. *et al.* Critical assessment of Belgian reimbursement dossiers of orphan drugs. *PharmacoEconomics* **29**, 883–893 (2011).

51. Persson, U. & Norlin, J. M. Multi-indication and Combination Pricing and Reimbursement of Pharmaceuticals: Opportunities for Improved Health Care through Faster Uptake of New Innovations. *Applied Health Economics and Health Policy* **16**, 157–165 (2018).

52. Takayama, A., Kobayashi, E., Nakamura, T. & Narukawa, M. Quantitative Assessment of Premium Rates for Clinical Usefulness in New Drug Price Calculation in Japan. *Therapeutic innovation & regulatory science* **51**, 582–588 (2017).

53. Barei, F. Value-added medicines: is there any room between market access challenges and 'patient centricity'? *Int. J. Pharm. Healthc. Mark.* **12**, 71–76 (2018).

54. Le Jeunne, C., Woronoff-Lemsi, M.-C., David, N. & de Sahb, R. La valeur ajoutée relative : quels outils pour l'évaluer ? *Therapie* **63**, 107–111 (2008).

55. Cañás, M., Buschiazzo, H. O. & Urtasun, M. A. Therapeutic value and price of the new pharmaceuticals commercialized in Argentina: Are they worth what they cost? *Salud colectiva* **15**, e1962 (2019).

56. Hansson, L. F., Norheim, O. F. & Ruyter, K. W. Equality, Explicitness, Severity, and Rigidity: The Oregon Plan Evaluated from a Scandinavian Perspective. *Journal of Medicine and Philosophy* **19**, 343–366 (1994).

57. Burkholder, R. Pricing and Value of Cancer Drugs. *JAMA oncology* **1**, 841–842 (2015).

58. McCabe, C., Claxton, K. & Tsuchiya, A. Orphan drugs and the NHS: should we value rarity? *BMJ* **331**, 1016–1019 (2005).

For peer review only - http://bmjopen.bmj.com/site/about/guidelines.xhtml

59. Philipson, T., Kamal-Bahl, S., Jena, A., Philipson, T. J. & Jena, A. B. Defining Value: The Need for a Longer, Broader View. *PharmacoEconomics* **35**, 669–672 (2017).

60. Walton, S. M., Basu, A., Mullahy, J., Hong, S. & Schumock, G. T. Measuring the Value of Pharmaceuticals in the US Health System. *PharmacoEconomics* **35**, 1–4 (2017).

61. Emanuel, E. J. *et al.* Drug Reimbursement Regulation in 6 Peer Countries. *JAMA Intern Med* **180**, 1510 (2020).

62. Vokinger, K. N. *et al.* Analysis of Launch and Postapproval Cancer Drug Pricing, Clinical Benefit, and Policy Implications in the US and Europe. *JAMA Oncol* **7**, e212026 (2021).

63. Moreau-Bachelard, C., Coquan, E. & Le Tourneau, C. Imputability of Adverse Events to Anticancer Drugs. *N Engl J Med* **380**, 1873–1874 (2019).

For peer review only

Appendix

Search Strategy
The search string contained (value or worth) or (“value based”) or (clinical* or medical* or therap* or cur* or pay* or drug* or pharmaceutical* or evaluat* or assess* or defin*). An additional operator was used so that there was a maximum of three words between the above terms and (valu* or worth). The second search string was (clinical* or medical* or therap* or cur* or patient*) and had an adjacent operator so that there was a maximum of three words benefit. The third search string was the following: ((drug* OR pharmac* OR medicine* OR medicat*) NEAR/20 pric*) OR AB= ((drug* OR pharmac* OR medicine* OR medicat*) NEAR/3 pric)).

Search Web of Science on the 18.12.2020

1. TI=((drug* OR pharmac* OR medicine* OR medicat*) NEAR/20 pric*) OR AB=((drug* OR pharmac* OR medicine* OR medicat*) NEAR/3 pric*)
   Indexes=SCI-EXPANDED, SSCI, A&HCI, CPCI-S, CPCI-SSH, BKCI-S, BKCI-SSH, ESCI,CCR-EXPANDED, IC Timespan=All years
2. TI=(value OR worth) OR TS=("value based") OR TS=((clinical* OR medical* OR therap* OR cur* OR pay* OR drug* OR pharmaceutical* OR evaluat* OR assess* OR defin*) NEAR/3 (valu* OR worth)) OR TS=((clinical* OR medical* OR therap* OR cur* OR patient*) NEAR/3 benefit)
   Indexes=SCI-EXPANDED, SSCI, A&HCI, CPCI-S, CPCI-SSH, BKCI-S, BKCI-SSH, ESCI, CCR-EXPANDED, IC Timespan=All years
3. #2 AND #1
   Indexes=SCI-EXPANDED, SSCI, A&HCI, CPCI-S, CPCI-SSH, BKCI-S, BKCI-SSH, ESCI,
   CCR-EXPANDED, IC Timespan=All years
4. #2 AND #1
   Refined by: LANGUAGES: (ENGLISH OR FRENCH OR GERMAN OR ITALIAN ORSPANISH)
   Indexes=SCI-EXPANDED, SSCI, A&HCI, CPCI-S, CPCI-SSH, BKCI-S, BKCI-SSH, ESCI, CCR-EXPANDED, IC Timespan=All year

Search Cochrane Library on the 18.12.2020

1. ((drug* OR pharmac* OR medicine* OR medicat*) NEAR/20 pric*):ti OR ((drug* OR pharmac* OR medicine* OR medicat*) NEAR/3 pric*):ab,kw
2. (value OR worth):ti OR 'value based':ti,ab,kw OR ((clinical* OR medical* OR therap* OR cur* OR pay* OR drug* OR pharmaceutical* OR evaluat* OR assess* OR defin*) NEAR/3 (valu* OR worth)):ti,ab,kw OR ((clinical* OR medical* OR therap* OR cur* OR patient*) NEAR/3 benefit):ti,ab,kw
3. #1 AND #2
4. Type a search term or use the S or MeSH buttons to compose

Search Scopus on the 18.12.2020

1. TITLE(value OR worth) OR TITLE-ABS-KEY("value based") OR TITLE-ABS-KEY((clinical* OR medical* OR therap* OR cur* OR pay* OR drug* OR pharmaceutical* OR evaluat* OR assess* OR defin*) W/3 (valu* OR worth)) OR TITLE-ABS-KEY((clinical* OR medical* OR therap* OR cur* OR patient*) W/3 benefit)
2. TITLE ((drug* OR pharmac* OR medicine* OR medicat*) W/20 pric*) OR ABS ((drug* OR pharmac* OR medicine* OR medicat*) W/3 pric*)) AND (TITLE (value OR worth) OR TITLE-ABS-KEY ("value based") OR TITLE-ABS-KEY ((clinical* OR medical* OR therap* OR cur* OR pay* OR drug* OR

For peer review only

pharmaceutical* OR evaluat* OR assess* OR defin*) W/3 (valu* OR worth)) OR TITLE-ABS-KEY ((clinical* OR medical* OR therap* OR cur* OR patient*) W/3 benefit)) AND (LIMIT-TO (LANGUAGE , "English") OR LIMIT-TO (LANGUAGE , "French") OR LIMIT-TO (LANGUAGE , "German") OR LIMIT-TO (LANGUAGE , "Spanish") OR LIMIT-TO (LANGUAGE , "Italian"))

3. (TITLE ((drug* OR pharmac* OR medicine* OR medicat*) W/20 pric*) OR ABS ((drug* OR pharmac* OR medicine* OR medicat*) W/3 pric*)) AND (TITLE (value OR worth) OR TITLE-ABS-KEY ("value based") OR TITLE-ABS-KEY ((clinical* OR medical* OR therap* OR cur* OR pay* OR drug* OR pharmaceutical* OR evaluat* OR assess* OR defin*) W/3 (valu* OR worth)) OR TITLE-ABS-KEY ((clinical* OR medical* OR therap* OR cur* OR patient*) W/3 benefit))
4. TITLE (value OR worth) OR TITLE-ABS-KEY ("value based") OR TITLE-ABS-KEY ((clinical* OR medical* OR therap* OR cur* OR pay* OR drug* OR pharmaceutical* OR evaluat* OR assess* OR defin*) W/3 (valu* OR worth)) OR TITLE-ABS-KEY ((clinical* OR medical* OR therap* OR cur* OR patient*) W/3
5. TITLE ((drug* OR pharmac* OR medicine* OR medicat*) W/20 pric*) OR ABS ((drug* OR pharmac* OR medicine* OR medicat*) W/3 pric*)

Search Regional Business News on the 18.12.2020

1. TI ((drug* OR pharmac* ORmedicine* OR medicat*) N20pric*) OR AB ((drug* OR pharmac* OR medicine* OR medicat*) N3 pric*)
2. TI (value OR worth) OR TI ("value based") OR TI ((clinical* OR medical* OR therap* OR cur* OR pay* OR drug* OR pharmaceutical* OR evaluat* OR assess* OR defin*) N3 (valu* OR worth)) OR TI ((clinical* OR medical* OR therap* OR cur* OR patient*) N3 benefit) OR AB ("value based") OR AB ((clinical* OR medical* OR therap* OR cur* OR pay* OR drug* OR pharmaceutical* OR evaluat* OR assess* OR defin*) N3 (valu* OR worth)) OR AB ((clinical* OR medical* ORtherap* OR cur* OR patient*) N3 benefit)
3. S1 AND S2

Search Medline on the 18.12.2020

1. (((MH "Drug Costs") OR (MH "Pharmaceutical Preparations+")) AND TX (pric*)) OR TI ((drug* OR pharmac* OR medicine* OR medicat*) N20 pric*) OR AB ((drug* OR pharmac* OR medicine* OR medicat*) N3 pric*
2. TI (value OR worth) OR TX ("value based") OR TX ((clinical* OR medical* OR therap* OR cur* OR pay* OR drug* OR pharmaceutical* OR evaluat* OR assess* OR defin*) N3 (valu* OR worth)) OR TX((clinical* OR medical* ORtherap* OR cur* OR patient*)N3 benefit)
3. S1 AND S2
4. S1 AND S2 with Limiters - Language:English, French, German, Italian, Spanish Expanders – Apply equivalent subjects Search modes - Find all mysearch term

Search Embase Session Results on the 18.12.2020

1. ('pharmacoeconomics'/exp OR 'drug'/exp) AND 'pric*':ti,ab,kw OR (((drug* OR pharmac* OR medicine* OR medicat*) NEAR/20 pric*):ti) OR (((drug* OR pharmac* OR medicine* OR medicat*) NEAR/3 pric*):ab,kw)
2. value:ti OR worth:ti OR 'value based':ti,ab,kw OR (((clinical* OR medical* OR therap* OR cur* OR pay* ORdrug* OR pharmaceutical* OR evaluat* OR assess* OR defin*)

For peer review only

NEAR/3 (valu* OR worth)):ti,ab,kw) OR (((clinical* OR medical* OR therap* OR cur* OR patient*) NEAR/3 benefit):ti,ab,kw)
3. #1 AND #2
4. #1 AND #2 AND [conference abstract]/lim
5. #1 AND #2 NOT [conference abstract]/lim
6. #1 AND #2 NOT [conference abstract]/lim AND ([english]/lim OR [french]/lim OR [german]/lim OR [italian]/lim OR [spanish]/lim)

EconLit on the 18.12.2020
1. TI ((drug* OR pharmac* OR medicine* OR medicat*) N20 pric*) OR AB ((drug* OR pharmac* OR medicine* OR medicat*) N3 pric*)
2. TI (value OR worth) OR TI ("value based") OR TI ((clinical* OR medical* OR therap* OR cur* OR pay* OR drug* OR pharmaceutical* OR evaluat* OR assess* OR defin*) N3 (valu* OR worth)) OR TI ((clinical* OR medical* OR therap* OR cur* OR patient*) N3 benefit) OR AB ("value based") OR AB ((clinical* OR medical* OR therap* OR cur* OR pay* OR drug* OR pharmaceutical* OR evaluat* OR assess* OR defin*) N3 (valu* OR worth)) OR AB ((clinical* OR medical* OR therap* OR cur* OR patient*) N3 benefit)
3. S1 AND S2

CINAHL on the 18.12.2020
1. ((MH "Drugs+") AND (TI (pric*) OR AB (pric*))) OR TI ((drug* OR pharmac* OR medicine* OR medicat*) N20 pric*) OR AB ((drug* OR pharmac* OR medicine* OR medicat*) N3 pric*)
2. TI (value OR worth) OR TI ("value based") OR TI ((clinical* OR medical* OR therap* OR cur* OR pay* OR drug* OR pharmaceutical* OR evaluat* OR assess* OR defin*) N3 (valu* OR worth)) OR TI ((clinical* OR medical* OR therap* OR cur* OR patient*) N3 benefit) OR AB ("value based") OR AB ((clinical* OR medical* OR therap* OR cur* OR pay* OR drug* ORpharmaceutical* OR evaluat*OR assess* OR defin*) N3(valu* OR worth)) OR AB ((clinical* OR medical* OR therap* OR cur* OR patient*)N3 benefit)
3. S1 AND S2
4. S1 AND S2

Search on Business Source Premier on 18.12.2020
1. DE "DRUG prices" OR DE "PHARMACEUTICAL reference pricing" OR TI ((drug* OR pharmac* OR medicine* OR medicat*) N20 pric*) OR AB ((drug* OR pharmac* OR medicine* OR medicat*) N3 pric*)
2. TI (value OR worth) OR TI ("value based") OR TI ((clinical* OR medical* OR therap* OR cur* OR pay* OR drug* OR pharmaceutical* OR evaluat* OR assess* OR defin*) N3 (valu* OR worth)) OR TI ((clinical* OR medical* OR therap* OR cur* OR patient*) N3 benefit) OR AB ("value based") OR AB ((clinical* OR medical* OR therap* OR cur* OR pay* OR drug* OR pharmaceutical* OR evaluat* OR assess* OR defin*) N3 (valu* OR worth)) OR AB ((clinical* OR medical* OR therap* OR cur* OR patient*) N3 benefit)
3. S1 AND S2

For peer review only

Preferred Reporting Items for Systematic reviews and Meta-Analyses extension for Scoping Reviews (PRISMA-ScR) Checklist

SECTION	ITEM	PRISMA-ScR CHECKLIST ITEM	REPORTED ON PAGE #
**TITLE**			
Title	1	Identify the report as a scoping review.	1, 2, 3
**ABSTRACT**			
Structured summary	2	Provide a structured summary that includes (as applicable): background, objectives, eligibility criteria, sources of evidence, charting methods, results, and conclusions that relate to the review questions and objectives.	2
**INTRODUCTION**			
Rationale	3	Describe the rationale for the review in the context of what is already known. Explain why the review questions/objectives lend themselves to a scoping review approach.	3
Objectives	4	Provide an explicit statement of the questions and objectives being addressed with reference to their key elements (e.g., population or participants, concepts, and context) or other relevant key elements used to conceptualize the review questions and/or objectives.	3
**METHODS**			
Protocol and registration	5	Indicate whether a review protocol exists; state if and where it can be accessed (e.g., a Web address); and if available, provide registration information, including the registration number.	Registration information on Prospero number CRD42021268174.
Eligibility criteria	6	Specify characteristics of the sources of evidence used as eligibility criteria (e.g., years considered, language, and publication status), and provide a rationale.	4
Information sources*	7	Describe all information sources in the search (e.g., databases with dates of coverage and contact with authors to identify additional sources), as well as the date the most recent search was executed.	3, 4
Search	8	Present the full electronic search strategy for at least 1 database, including any limits used, such that it could be repeated.	3,4
Selection of sources of evidence†	9	State the process for selecting sources of evidence (i.e., screening and eligibility) included in the scoping review.	3, 4
Data charting process‡	10	Describe the methods of charting data from the included sources of evidence (e.g., calibrated forms or forms that have been tested by the team before their use, and whether data charting was done independently or in duplicate) and any processes for obtaining and confirming data from investigators.	3,4

For peer review only - http://bmjopen.bmj.com/site/about/guidelines.xhtml

SECTION	ITEM	PRISMA-ScR CHECKLIST ITEM	REPORTED ON PAGE #
Data items	11	List and define all variables for which data were sought and any assumptions and simplifications made.	4
Critical appraisal of individual sources of evidence§	12	If done, provide a rationale for conducting a critical appraisal of included sources of evidence; describe the methods used and how this information was used in any data synthesis (if appropriate).	NA
Synthesis of results	13	Describe the methods of handling and summarizing the data that were charted.	4
**RESULTS**			
Selection of sources of evidence	14	Give numbers of sources of evidence screened, assessed for eligibility, and included in the review, with reasons for exclusions at each stage, ideally using a flow diagram.	Figure 1, page 11
Characteristics of sources of evidence	15	For each source of evidence, present characteristics for which data were charted and provide the citations.	Tables 2-4
Critical appraisal within sources of evidence	16	If done, present data on critical appraisal of included sources of evidence (see item 12).	NA
Results of individual sources of evidence	17	For each included source of evidence, present the relevant data that were charted that relate to the review questions and objectives.	Tables 2-4
Synthesis of results	18	Summarize and/or present the charting results as they relate to the review questions and objectives.	5
**DISCUSSION**			
Summary of evidence	19	Summarize the main results (including an overview of concepts, themes, and types of evidence available), link to the review questions and objectives, and consider the relevance to key groups.	8
Limitations	20	Discuss the limitations of the scoping review process.	9
Conclusions	21	Provide a general interpretation of the results with respect to the review questions and objectives, as well as potential implications and/or next steps.	8, 9, 10
**FUNDING**			
Funding	22	Describe sources of funding for the included sources of evidence, as well as sources of funding for the scoping review. Describe the role of the funders of the scoping review.	1

JBI = Joanna Briggs Institute; PRISMA-ScR = Preferred Reporting Items for Systematic reviews and Meta-Analyses extension for Scoping Reviews.
* Where *sources of evidence* (see second footnote) are compiled from, such as bibliographic databases, social media platforms, and Web sites.
† A more inclusive/heterogeneous term used to account for the different types of evidence or data sources (e.g., quantitative and/or qualitative research, expert opinion, and policy documents) that may be eligible in a scoping review as opposed to only studies. This is not to be confused with *information sources* (see first footnote).

For peer review only - http://bmjopen.bmj.com/site/about/guidelines.xhtml

‡ The frameworks by Arksey and O'Malley (6) and Levac and colleagues (7) and the JBI guidance (4, 5) refer to the process of data extraction in a scoping review as data charting.
§ The process of systematically examining research evidence to assess its validity, results, and relevance before using it to inform a decision. This term is used for items 12 and 19 instead of "risk of bias" (which is more applicable to systematic reviews of interventions) to include and acknowledge the various sources of evidence that may be used in a scoping review (e.g., quantitative and/or qualitative research, expert opinion, and policy document).

From: Tricco AC, Lillie E, Zarin W, O'Brien KK, Colquhoun H, Levac D, et al. PRISMA Extension for Scoping Reviews (PRISMAScR): Checklist and Explanation. Ann Intern Med. 2018;169:467–473. doi: 10.7326/M18-0850.

For peer review only

BMJ Open

Defining ‘therapeutic value’ of medicines: a scoping review

Journal:	*BMJ Open*
Manuscript ID	bmjopen-2023-078134.R1
Article Type:	Original research
Date Submitted by the Author:	25-Oct-2023
Complete List of Authors:	Glaus, Camille ; University of Zurich, Rechtswissenschaftliches Institut<br>Kloeti, Andrina; University of Zurich<br>Vokinger, Kerstin; University of Zurich, Institute of Law; Harvard Medical School, Program on Regulation, Therapeutics, and Law
**Primary Subject Heading**:	Health policy
Secondary Subject Heading:	Health policy
Keywords:	Health policy < HEALTH SERVICES ADMINISTRATION & MANAGEMENT, MEDICAL ETHICS, Health economics < HEALTH SERVICES ADMINISTRATION & MANAGEMENT

SCHOLARONE™
Manuscripts

I, the Submitting Author has the right to grant and does grant on behalf of all authors of the Work (as defined in the below author licence), an exclusive licence and/or a non-exclusive licence for contributions from authors who are: i) UK Crown employees; ii) where BMJ has agreed a CC-BY licence shall apply, and/or iii) in accordance with the terms applicable for US Federal Government officers or employees acting as part of their official duties; on a worldwide, perpetual, irrevocable, royalty-free basis to BMJ Publishing Group Ltd ("BMJ") its licensees and where the relevant Journal is co-owned by BMJ to the co-owners of the Journal, to publish the Work in this journal and any other BMJ products and to exploit all rights, as set out in our licence.

The Submitting Author accepts and understands that any supply made under these terms is made by BMJ to the Submitting Author unless you are acting as an employee on behalf of your employer or a postgraduate student of an affiliated institution which is paying any applicable article publishing charge ("APC") for Open Access articles. Where the Submitting Author wishes to make the Work available on an Open Access basis (and intends to pay the relevant APC), the terms of reuse of such Open Access shall be governed by a Creative Commons licence – details of these licences and which Creative Commons licence will apply to this Work are set out in our licence referred to above.

Other than as permitted in any relevant BMJ Author's Self Archiving Policies, I confirm this Work has not been accepted for publication elsewhere, is not being considered for publication elsewhere and does not duplicate material already published. I confirm all authors consent to publication of this Work and authorise the granting of this licence.

For peer review only

Defining 'therapeutic value' of medicines: a scoping review

Camille EG Glaus, BSc, JD[1], Andrina Kloeti, MMed[1], Kerstin N Vokinger, MD, JD, PhD[1]

Affiliations

[1] Institute of Law, University of Zurich, Zurich, Switzerland.

Correspondence to:

Prof Kerstin N Vokinger, Institute of Law, University of Zurich, Ramistrasse 74, 8001 Zurich, Switzerland

Email: Lst.vokinger@rwi.uzh.ch

Word count: 2,670

For peer review only

Abstract

Objectives: In recent years, discussions on the importance and scope of therapeutic value of new medicines have intensified, stimulated by the increase of prices and number of medicines entering the market. This study aims to perform a scoping review identifying factors contributing to the definition of the therapeutic value of medicines.

Design: Scoping review.

Data sources: We searched the MEDLINE, CINAHL, Embase, Business Source Premier, EconLit, Regional Business News, Cochrane, Web of Science, Scope and Pool databases through December 2020 in English, German, French, Italian and Spanish.

Eligibility criteria: Studies that included determinants for the definition of therapeutic value of medicines were included.

Data extraction and synthesis: Data was extracted using the mentioned data sources mentioned. Two reviewers independently screened and analyzed the articles. Data were analyzed from April 2021 to May 2022.

Results: Of the 1883 studies screened, 51 were selected and the identified factors contributing to the definition of therapeutic value of medicines were classified in three categories: Patient perspective, public health perspective, and socio-economic perspective. More than three-quarters of the included studies were published after 2014, with the majority of the studies focusing on either cancer disorders (14/51, 27.5%) or rare diseases (11/51, 21.6%). Frequently mentioned determinants for value were quality of life, therapeutic alternatives and side effects (all patient perspective), prevalence/incidence and clinical endpoints (all public health perspective), and costs (socio-economic perspective).

Conclusions: Multiple determinants have been developed to define the therapeutic value of medicines, most of them focusing on cancer disorders and rare diseases. Considering the relevance of value of medicines to guide patients and physicians in decision-making as well as policymakers in resource allocation decisions, a development of evidence-based factors for the definition of therapeutic value of medicines is needed across all therapeutic areas.

For peer review only

For peer review only - http://bmjopen.bmj.com/site/about/guidelines.xhtml

Strengths and limitations of this study

- This scoping review was based on a comprehensive search and articles were screened and analyzed by two independent authors.
- Potential studies could be missed due to the heterogeneity of terms.
- Another categorization of value determinants is possible outside of therapeutics.

Introduction

In recent years, discussions on the importance of value of new medicines have intensified, stimulated by the increase of prices and number of medicines entering the market.(1) Several countries with price regulations (for example Germany or France) have tasked health technology agencies to assess the added therapeutic value of new medicines as a basis for price negotiation.(2) Also medical associations developed frameworks that enable the value assessment of medicines with the goal to support physicians and patients in their decision-making. For example, the European Society for Medical Oncology (ESMO) created the ESMO-magnitude of clinical benefit scale, and the American Society of Clinical Oncology (ASCO) implemented the ASCO-value framework specifically for cancer medicines.(3,4) Additionally, non-profit organizations such as the Institute for Clinical and Economic Review (ICER) emerged with the goal of conducting and disseminating comparative effectiveness evaluations to, among other things, encourage fair pricing.(5)

Studies indicate that only a fraction of new medicines provide high added therapeutic value when applying the therapeutic rating of health technology agencies or medical associations.(6,7,8) However, value of medicines can vary depending on the underlying understanding of value.(9,10)

The importance of the determinants contributing to the assessment of therapeutic value of medicines will further increase in the upcoming years given the growth of high costs of medicines that threaten healthcare budgets across countries. To support ongoing discussions on how to define value, we undertook a scoping review of the literature to identify the determinants used to assess the therapeutic value of medicines.

Methods

<u>Selection criteria</u>

This scoping review was conducted using the PRISMA guidelines for scoping reviews. In December 2020, the databases Medline (PubMed), CINAHL, EMBASE, Business Source Premier, EconLit, Regional Business News, Cochrane, Web of Science, Scope and Pool

For peer review only - http://bmjopen.bmj.com/site/about/guidelines.xhtml

were searched for studies published until December 2020 in English, German, French, Italian and Spanish.

Search strategy

The search string contained (value or worth) or ("value based") or (clinical* or medical* or therap* or cur* or pay* or drug* or pharmaceutical* or evaluat* or assess* or defin*). An additional operator was used so that there was a maximum of three words between the above terms and (valu* or worth). The second search string was (clinical* or medical* or therap* or cur* or patient*) and had an adjacent operator so that there was a maximum of three words benefit. The third search string was the following: ((drug* OR pharmac* OR medicine* OR medicat*) NEAR/20 pric*) OR AB= ((drug* OR pharmac* OR medicine* OR medicat*) NEAR/3 pric)). A more detailed explanation of the search strategy can be found in the supplementary file.

Screening protocol

Two authors (CEGG, AK) independently screened the articles following a fixed protocol. They both screened all abstracts and titles and titles based on the inclusion criteria. They then compared their results and established a list with the non-congruent results. Disagreement was resolved in discussion with the last author (KNV). The same procedure was followed for the screening of the full texts (**Table 1**).

Study selection

For studies that appeared from their title or abstract to discuss determinants of the therapeutic value of medicines, full articles related to these titles were obtained and screened. Studies were included if they proposed a definition and/or one or more determinants of therapeutic value of medicines. We excluded studies that provided information about the value of specific medicines but did not include specific information on determinants of value. We also excluded studies that focused on related topics but did not directly address the determinants of therapeutic value, such as value-based pricing.

Analysis

The first author (CEGG) reassessed the identified results and divided them in three categories: patient perspective, public health perspective and socio-economic perspective. This decision was discussed with the last author (KNV). Studies that matched more than one category were included separately in each category. The studies were assessed from April 2021 to May 2022. This analysis was descriptive neither the veracity nor the justifiability of the results were assessed.

For peer review only - http://bmjopen.bmj.com/site/about/guidelines.xhtml

The study did not require institutional review board approval because the data were based on publicly available information. The study adheres with the EQUATOR Reporting Guidelines. Detailed methods are presented in the Supplement.

Patient and public involvement

None.

Results

The search strategy generated 1833 records, of which we identified 442 for full review. 44 unique papers were included. Reference mining led us to 7 additional articles that met our criteria, resulting in a total of 51 articles for the final sample. **Figure 1** provides a flow diagram of the search results.

The dates of the articles range between 1996 and 2020, although fewer than one-quarter (11/50, 22%) were published prior 2014.

Of the 51 studies, most determinants of value focused on cancer medicines (14/51, 27.5%) or medicines targeting orphan diseases (11/51, 21.6%). 5/51 (9.8%) were for other therapeutic areas (such as chronic diseases(11) or neurologic diseases(12) and 21/51 (41.2%) were unspecified.

Some studies outlined a holistic approach for the assessment of value of medicines. For example, Schnipper and colleagues outlined that value is generally accepted as a measure of outcomes achieved per monetary expenditure.(3) However, in most cases the studies did not incorporate a holistic approach, but rather focused on a perspective (e.g., patient perspective) and on specific determinants (**Tables 2–4**). We divided the different determinants of value in three categories. Some determinants can be classified in more than one category. For example, we classified the determinant 'effectiveness' under the public policy perspective, alternatively it could also fall into the patient perspective.

Value of medicines: patient perspective

We identified 48 articles that matched this category, focusing on the following aspects: Quality of life (31 studies)(3,4,11-39), burden of disease (including severity of the disease, unmet medical need, 39 studies)(3,11,13,15-17,19-22,24-32,34-37,39-54) and convenience (e.g., administration route, 16 studies)(3,11-13,15,22,24,28,29,31,35,36,49,52,53,55) (**Table 2**).

Quality of life

For peer review only

Many studies acknowledged the importance of quality of life when assessing the value of medicines.(28,36) However, most studies did not offer a clear or unified definition for this determinant. Some studies specified quality of life as the impact of the treatment on the physical and mental abilities.(3,30,31,35) Another study highlighted that quality of life for cancer patients should be assessed in a questionnaire that addresses four categories: social well-being, emotional well-being, memory and need of a caregiver.(27) And a further study emphasized that quality of life is inherent to the characteristics of the individual patient.(11)

Burden of disease

A unified definition could also not be identified for the value determinant burden of disease. For example, different definitions were applied for the determinant "severity". One study stated that "severity of the disease relates to the condition's degree of seriousness in response to mortality and morbidity-derived disability […] or the expected remaining life years adjusted for their quality of life.(56) Another study defined severity more broadly as the "overall impact of a problem on an individual."(25) This was specified in other studies, which stated that the severity of a disease included physical and mental aspects of the disease.(25,26,35)

Unmet medical need was a further frequently mentioned determinant for the evaluation of value of medicines, especially in the context of orphan diseases.(26,35,37,43,46,47) Unmet medical need was often defined as the therapeutic options for the patient or the number of available medicines for the specific disease.(21,26,35,41,42,47) Some studies underlined that the benefits of alternative medicines should also be considered.(35,47) Medicines targeting a disease with no alternative treatment option available should be granted a higher value.(47)

<ins>Value of medicines: public health perspective</ins>

49 studies in our study cohort focused on this perspective when assessing value of medicines. We identified the following clusters: Epidemiological endpoints (e.g., size of population, 13 studies)(14,15,24,26,35,37,40,42,45,46,50,57,58) and clinical endpoints (e.g., safety, efficacy or evidence, 45 studies)(3,4,12,13,15-25,27-33,35-37,39-42,44-55,57-60) (**Table 3**).

Epidemiological endpoints

Multiple studies referred to epidemiological endpoints (for example, incidence or prevalence), as an important aspect for the evaluation of value of medicine (10 studies).(15,24,26,35,37,42,45,46,50,57) Some studies argued that the rarity of a disease increases the value of the medicines targeting those diseases,(25,26,35) while others stated

For peer review only

that this approach is unethical.(24,58) Some studies also argued that taking into account epidemiological endpoints would be unethical since that would unjustifiably increase the treatment value of certain diseases compared to others.(24,58)

Clinical endpoints

Many studies considered outcomes of clinical trials, such as safety or effectiveness as core determinants for the evaluation of value of medicines.(3,4,12-20,22-33,35,37,39-42,44-55,58-60), even more so in more recent studies.(16,22,24,32) Trial outcomes are influenced by the understanding of the disease,(37) the stage of the disease (the benefit of medicines targeting the treatment of advanced-stage diseases may be more modest compared to those applied in a curative setting),(3,16,31,38) the duration of the study (some medicines result in a short tumor response and in case of a short study duration the long-term effects may be unclear),(16) the size of the targeted population evaluated,(26,37,47,50) the study design,(24,26) the subjects of the study and the potential resulting heterogeneity in medicine response.(11,16,22,27,32,60) Concerns with regard to the lack of evidence was particularly raised for medicines targeting orphan diseases and cancer medicines.(26,37,47,50) Value of medicines based on outcomes of clinical trials may change over a medicine's lifecycle depending on, among other things, new evidence provided in further trials,(16,32,58) additional indications that increase the aggregated value to society,(13,24,59) and outcomes in long-term studies.(16)

Side effects and toxicity were other important determinants frequently highlighted as determinants for the assessment of value of medicines.(3,4,15,16,21-25,27,28,30,31,33,36,37,39,48,50,59) Multiple studies specified side effects and toxicity as tolerability, discontinuation or complications.(12,13,21,21,24,35) In the same context, several authors stressed that compliance to a medicine is an important element of value.(13,20,22,25,28-30,32,33,35,42,53,54)

<u>Value of medicines: socio-economic perspective</u>

We included 46 studies in the study cohort. We identified the following clusters: Economic burden (e.g., treatment costs, non-treatment costs, and willingness to pay, 21 studies)(3,11-16,22,24,25,28,29,31-34,36,44,49,51,60, innovation (e.g., mechanism of action or spill-over effect, latter includes, for example, benefits that may evolve due to the treatment of the disease, 26 studies)(11,13,17,19,22,24-26,32,34-36,40-45,49,51-55,59,60), and broader social impact (e.g., equity, 23 studies)(3,15-17,24,27,28,31,33,34,36-38,42,45,49,50,53-55,58-60) (**Table 4**).

Economic burden

For peer review only - http://bmjopen.bmj.com/site/about/guidelines.xhtml

Treatment costs and non-treatment costs (e.g., caregiver costs, lost work productivity) were considered by multiple studies as important determinants to appraise value of medicines.(3,11-16,22,24,25,29,31-34,36,42,44,49,60) Non-treatment costs include also the financial burden for society, which was identified as a relevant determinant for the assessment of value of medicines.(11,24,33,37,38,49,50)

Some studies defined value as the willingness to pay for a medicine.(11,14,15,28,30,32,33,51) One study underlined the subjectivity of this criteria, highlighting that, for example, patients may have another willingness compared to physicians and that the willingness to pay can also vary among patients or physicians.(11)

Innovation

Many studies argued that innovation is another core element when assessing value of medicines.(11,13,17,19,22,24-26,32,34-36,40-45,49,51-55,59,60) There was not one unified definition for innovation. Examples included the medicine's novel mechanism of action,(13,19,24,42,51,52,54,55) its effectiveness and side effects,(48) its spill-over effect (understood as the gained knowledge during the development of the medicine that serves as a basis for the development of further medicines),(13,24,34,36,41,45,59,60) or the route of medicine administration.(13)

Other identified determinants for the evaluation of value of medicines were the research and development costs,(26,33,35) or the level of complexity of the medicine.(26,35,43) Another study explicitly stated that research and development costs should not be considered for the assessment of value, arguing that value should only reflect the direct benefits for the patient.(14)

Social impact

Some studies considered the broader social impact of medicines as important elements for the assessment of value. It includes general ethical principles, such as altruism towards the poor,(59) discrimination,(17,49) fairness,(40) or equity.(3,31,34,37,38,42,48,58) It also comprises other societal determinants such as prevention,(24,25,38) and the relief of family members of emotionally and physically demanding responsibilities.(25,27,28,37,59)

Discussion

More than three quarter of the included studies were published after 2014, with the majority of the studies focusing on either cancers or rare diseases. A major amount of the included

For peer review only - http://bmjopen.bmj.com/site/about/guidelines.xhtml

studies highlighted only specific aspects of value of medicines that we classified in three categories: Patient perspective, public health perspective, and socio-economic perspective.

Our study findings have implications for the ongoing discussions around value of medicines. Value assessment of medicines support, for example, patients and physicians in decision-making in selecting the best treatment for an individual patient, or health technology agencies and policymakers in resource allocation and reimbursement decisions.(3,61) Given the importance of value assessment of medicines, our study findings suggest that more analysis and discussions are indicated to develop an evidence-based definition for and understanding of value. The identified determinants in this study for the value of medicines could serve as a basis in this regard. Frequently mentioned determinants for value were quality of life, therapeutic alternatives and side effects (all patient perspective), prevalence/incidence and clinical endpoints (all public health perspective), and costs (socio-economic perspective). The specific determinants for the value assessment of medicines may vary depending on the country, health care system, therapeutic area and patient population.(3)

Value determinants, such as a medicine's evidence, clinical outcomes or its impact on quality of life can change over time based either on new findings from additional clinical trials or real-world evidence or in comparison to new medicines entering the market for the same indication.(59) These study findings suggest that the value of medicines should not be understood statically but rather dynamically, which is consistent with the approach of HTA bodies or agencies that re-assess value and prices of medicines either on a regular basis (e.g., every three years in Switzerland) or if new evidence from clinical trials or real-world data is provided (e.g., Germany).

Many of the studies in the study cohort were published in recent years, with the majority of the studies focusing on either cancers (14/51, 27.5%) or rare diseases (11/51, 21.6%). The focus on these medicines might be explained by their high treatment prices, their potentially severe adverse events and the increasing amount of medicines in these therapeutic areas entering the market.(62,63) Furthermore, medical associations such as ASCO and ESMO developed value frameworks specifically for cancer medicines. By contrast, for example, only one included study focused on the determinants of value specifically for diabetic medicines.(18) Also other therapeutic areas, such as psychiatry or neurology, are not or only marginally addressed in the included studies. A stronger focus on the factors of value of medicines in general and for therapeutic areas other than cancer and rare diseases is indicated.

<u>Limitations</u>

For peer review only - http://bmjopen.bmj.com/site/about/guidelines.xhtml

This study has limitations. Due to the broadness of the topic, it is possible that the study cohort is not exhaustive. Our multilayered search strategy allowed us to screen a wide range of studies, leaving us confident that we reached thematic saturation for main outcomes. However, we focused only on determinants defining the therapeutic value of medicines and excluded the rich literature on related topics such as “value in healthcare” or “health care services.” We excluded reports and protocols of HTA bodies, which may also provide important insights on the determinants of therapeutic value. This would be interesting to assess in a follow-up study, however, it would need the consultation of other databases compared to this literature search. Furthermore, we only focused on studies published in English, German, French, Italian or Spanish. Thus, it is possible that we did not include relevant studies published in other languages. Lastly, another categorization of value determinants is possible. Some determinants fulfill more than one category. In such cases, we categorized the determinant in the category that we consider as more adequate. For example, “quality of life” is not only relevant from a patient perspective, but is also a public health consideration.

Conclusions

Multiple determinants have been developed to define the therapeutic value of medicines. Most definitions and determinants identified for the assessment of therapeutic value of medicines were developed for cancer disorders and rare diseases. Considering the relevance of value of medicines to guide patients and physicians in decision-making as well as policymakers in resource allocation decisions, a development of evidence-based factors for the definition of therapeutic value of medicines is needed, addressing also diseases outside cancer disorders and orphan diseases.

For peer review only

For peer review only - http://bmjopen.bmj.com/site/about/guidelines.xhtml

Table 1. Criteria for study selection

Inclusion	Exclusion
• Terms “value”, “benefit” if determinants of value of medicine or as argument for pricing<br>• New tool/concept or proposed adoption of already existing tool/concept<br>• Tool/concepts by countries/state institutions if these countries/state institutions developed/adapted them or if they are unclear<br>• Journals<br>• Conference papers	• No direct link to “value”, “HTA”, “benefit” (e.g. accessibility, innovation)<br>• Application of already existing toolsconcepts<br>• Tool/concept by countries/state institutions if they only apply an already existing tool/concept<br>• Complementary medicines<br>• Biosimilars<br>• Generics<br>• Drugs (e.g. randomized clinical trials)<br>• Contracting and insurance design<br>• News articles

Table 2. Identified determinants for the assessment of value of medicines from the patient perspective

Patient perspective				
**Category**	**Medical speciality**			
	**Oncology (N = 14)**	**Orphan disease (N = 11)**	**Other (N = 5)**	**Not specified (N = 21)**
**Quality of life**	3, 4, 13, 15, 16, 21, 23, 27, 29, 31	17, 26, 35, 37	11, 12, 18, 30	14, 19, 20, 22, 24, 25, 28, 32, 33, 34, 36, 38, 39
**Burden of disease**				
• General definition	29, 41, 42	17, 37, 43, 44, 46, 47	30	19, 24, 32, 36, 49, 54
• Severity of disease, symptoms	3, 13, 15, 16, 21, 27, 31, 40, 41	17, 26, 35, 37, 43–47, 50	11, 30, 48	24, 25, 28, 32, 34, 36, 39, 49, 51, 52, 54
• Unmet medical need, alternatives	15, 21, 29, 40–42	26, 35, 37, 43, 46, 47	30	19, 20, 22, 24, 28, 32, 49, 52–54
• Others: patient experience, fear of contagion, daily activity,	3, 16, 21, 27, 29, 31, 42	37	11	19, 20, 25, 36, 38, 49, 53

For peer review only - http://bmjopen.bmj.com/site/about/guidelines.xhtml

reaching a landmark life event, dignity				
**Convenience**				
• Posology, route of administration, instructions, comfort	3, 13, 15, 29, 31	35	11, 12	22, 24, 28, 36, 49, 52, 53, 55

Note: The numbers listed in the medical specialty match the references. Studies that address multiple categories for the definition of value of medicines were listed for each category separately.

Table 3. Identified determinants for the assessment of value of medicines from the public health perspective

**Public health perspective**				
**Category**	**Medical specialty**			
	**Oncology (N = 14)**	**Orphan disease (N = 11)**	**Other (N = 5)**	**Not specified (N = 21)**
**Epidemiological endpoints**				
• Prevalence, incidence, rarity	15, 40, 42, 57	26, 35, 37, 45, 46, 50		24
**Clinical endpoints and evidence**				
• Safety	3, 16, 29, 31	35, 44	12, 48	19, 20, 22, 24, 28, 32, 33, 53, 55
• Efficacy	13, 15, 16, 29	35, 44, 50		19, 20, 22, 24, 32, 33, 52-55
• Effectiveness	3, 13, 15, 31, 40, 42	26, 35, 37	18, 30, 48	14, 20, 25, 33, 51, 53, 59, 60
• Clinical benefits	3, 4, 16, 23, 27, 29, 31, 41	17, 26, 35, 45–47, 58	30	28, 39, 49, 51, 54, 59
• Side effects, toxicity	3, 4, 13, 15, 16, 21, 23, 27, 31	37	48, 30	22, 24, 25, 28, 33, 36, 39
• Compliance, discontinuation, tolerability	13, 29	35	30	20, 22, 25, 28, 32, 33, 42, 53, 54
• Certainty, evidence	3, 16, 31, 42, 57	26, 37, 47, 50, 58		19, 22, 24, 25, 32, 36, 39, 51, 52, 60

Note: The numbers listed in the medical specialty match the references. Studies that address multiple categories for the definition of value of medicines were listed for each category separately.

Table 4. Identified determinants for the assessment of value of medicines from the socioeconomic perspective

Socio-economic perspective				
**Category**	**Medical specialty**			
	**Oncology (N = 14)**	**Orphan disease (N = 11)**	**Other (N = 5)**	**Not specified (N = 21)**
**Economic burden**				
• Treatment costs	3, 13, 29, 31	44	11, 12	22, 24, 33, 34, 36
• Non-treatment costs	3, 15, 16, 29, 31, 42	44	11	14, 22, 24, 25, 32, 34, 36, 49, 60
• Willingness to pay	15		11, 30	14, 28, 32, 33, 51
**Innovation**				
• Innovation	40, 41	17, 44	11	22, 24, 25, 32, 36, 49, 52, 53
• Spill-over in research	13, 41	45		24, 34, 36, 49, 59, 60
• Mechanism of action, effectiveness, route of administration	13, 42		48	19, 24, 51, 52, 54, 55
• Others: research undertaken, unique indication, complexity of innovation, techological considerations	41	26, 35, 43		33, 54, 59
**Broader social impact**				
• Social value				28, 33, 36, 49, 59
• Altruism				59
• Discrimination		17		49
• Equity, fairness	3, 31, 40, 42	37, 58	48	34, 38
• Family benefit	27	37		25, 28, 59
• Others: prevention, risk reduction, feasability (adoption), public health benefits	15, 16	45, 50		24, 25, 38, 53, 54, 60

Note: The numbers listed in the medical specialty match the references. Studies that address multiple categories for the definition of value of medicines were listed for each category separately.

For peer review only

For peer review only - http://bmjopen.bmj.com/site/about/guidelines.xhtml

Funding: This study was funded by the Swiss National Science Foundation (SNSF, grant number 194607).

Competing interests: The authors declare no competing interests.

Data availability statement: All the data included in the study are publicly available. Data are available upon request.

Contributors: CEEG contributed to the data collection, data analysis, data interpretation, first rudimentary draft, and revision of the manuscript. AK contributed to the data collection and data analysis. KNV contributed to the acquisition of funding, conception, data collection, data interpretation, first draft, and revision of the manuscript. All authors have access to all the data in the manuscript.

Ethics approval statement: The study did not require institutional review board approval because the data were based on publicly available information and no patients were involved.

For peer review only

References

1. Darrow, J. J., Avorn, J. & Kesselheim, A. S. FDA Approval and Regulation of Pharmaceuticals, 1983-2018. *JAMA* **323**, 164 (2020).
2. Vokinger, K. N. *et al.* Prices and clinical benefit of cancer drugs in the USA and Europe: a cost-benefit analysis. *The Lancet. Oncology* **21**, 664–670 (2020).
3. Schnipper, L. E. *et al.* American Society of Clinical Oncology Statement: A Conceptual Framework to Assess the Value of Cancer Treatment Options. *JCO* **33**, 2563–2577 (2015).
4. Cherny, N. I. *et al.* A standardised, generic, validated approach to stratify the magnitude of clinical benefit that can be anticipated from anti-cancer therapies: the European Society for Medical Oncology Magnitude of Clinical Benefit Scale (ESMO-MCBS). *Annals of Oncology* **26**, 1547–1573 (2015).
5. ICER. Institute For Clinical And Economic Review.
6. Hwang, T. J., Ross, J. S., Vokinger, K. N. & Kesselheim, A. S. Association between FDA and EMA expedited approval programs and therapeutic value of new medicines: retrospective cohort study. *BMJ* m3434 (2020) doi:10.1136/bmj.m3434.
7. Hwang, T. J., Vokinger, K. N. & Kesselheim, A. S. New Treatments for Migraine—Therapeutic Ratings and Comparative Coverage in the US, Canada, and Europe. *JAMA Intern Med* **182**, 101 (2022).
8. Vokinger, K. N., Hwang, T. J., Glaus, C. E. G. & Kesselheim, A. S. Therapeutic Value Assessments of Novel Medicines in the US and Europe, 2018-2019. *JAMA Netw Open* **5**, e226479 (2022).
9. Hu, I., Zheng, J. & Hu, X. H. Comparison Of Health Technology Assessment (Hta) Rankings By German And French Hta Agencies. *Value in Health* **18**, A96–A97 (2015).
10. Cherny, N. I. *et al.* Comparative Assessment of Clinical Benefit Using the ESMO-Magnitude of Clinical Benefit Scale Version 1.1 and the ASCO Value Framework Net Health Benefit Score. *JCO* **37**, 336–349 (2019).

For peer review only - http://bmjopen.bmj.com/site/about/guidelines.xhtml

11. Koh, C. Y. C. & Seager, T. P. Value-Based Pharmaceutical Pricing From the Patient Perspective Could Incentivize Innovation. *Pharmaceutical Medicine* **31**, 149–153 (2017).

12. Crowell, J. L. & Burns, T. M. Rising Drug Costs for Neurologic Diseases. *Continuum (Minneapolis, Minn.)* **26**, 1392–1406 (2020).

13. Angelis, A. *et al.* Multiple Criteria Decision Analysis for HTA across four EU Member States: Piloting the Advance Value Framework. *Social Science and Medicine* **246**, (2020).

14. Cohen, J. T., Anderson, J. E. & Neumann, P. J. Three Sets of Case Studies Suggest Logic and Consistency Challenges with Value Frameworks. *Value in Health* **20**, 193–199 (2017).

15. Doyle, J. J., Hawryluk, E., Niemira, J. & Wood, B. Evidence-Based Valuation in Oncology:: Lessons Learned from a Case Study. *Therapeutic innovation & regulatory science* **53**, 403–411 (2019).

16. Jönsson, B. & Wilking, N. New cancer drugs in Sweden: Assessment, implementation and access. *Journal of Cancer Policy* **2**, 45–62 (2014).

17. Kiernan, F. The future of pharmacoeconomic policy - does value-based pricing really have a role? *Journal of Pharmaceutical Health Services Research* **7**, 5–9 (2016).

18. Cheng, W. H., Gaudette, É. & Goldman, D. P. PCSK9 Inhibitors Show Value for Patients and the US Health Care System. *Value in Health* **20**, 1270–1278 (2017).

19. Abedi, S. The Payer Perspective. *Pharmaceutical Executive Global Digest* **35**, 40–41 (2015).

20. DeKoven, M., Hazard, E. H., Goldberg, E. & Pokras, S. Got value? Determine it. Demonstrate it. Communicate it. Realise it. *Journal of Commercial Biotechnology* **14**, 299–306 (2008).

For peer review only

21. Fonseca, R. & Hinkel, J. Value and Cost of Myeloma Therapy-We Can Afford It. *American Society of Clinical Oncology educational book. American Society of Clinical Oncology. Annual Meeting* **38**, 647–655 (2018).

22. Gregson, N., Sparrowhawk, K., Mauskopf, J. & Paul, J. Pricing medicines: theory and practice, challenges and opportunities. *Nature reviews. Drug discovery* **4**, 121–130 (2005).

23. Cherny, N. I. *et al.* ESMO-Magnitude of Clinical Benefit Scale version 1.1. *Annals of Oncology* **28**, 2340–2366 (2017).

24. Angelis, A. & Kanavos, P. Multiple Criteria Decision Analysis (MCDA) for evaluating new medicines in Health Technology Assessment and beyond: The Advance Value Framework. *Social science & medicine (1982)* **188**, 137–156 (2017).

25. Dionne, F., Mitton, C., Dempster, B. & Lynd, L. D. Developing a multi-criteria approach for drug reimbursement decision making: an initial step forward. *Journal of population therapeutics and clinical pharmacology = Journal de la therapeutique des populations et de la pharmacologie clinique* **22**, e68–e77 (2015).

26. Hughes-Wilson, W., Palma, A., Schuurman, A. & Simoens, S. Paying for the Orphan Drug System: break or bend? Is it time for a new evaluation system for payers in Europe to take account of new rare disease treatments? *Orphanet journal of rare diseases* **7**, 74 (2012).

27. Lorgelly, P. *et al.* Outcome-Based Payment Schemes: What Outcomes Do Patients with Cancer Value? *The patient* **13**, 599–610 (2020).

28. Puig-Junoy, J. & Peiró, S. From the therapeutic utility to the added therapeutic value and the incremental cost-effectiveness ratio. *Revista Espanola de Salud Publica* **83**, 59–70 (2009).

29. Rothenberg, M. L., McCarthy, J. & Holmes, P. Price, Cost, and Value of Cancer Medicines: A Pharmaceutical Industry Perspective. *Cancer journal (Sudbury, Mass.)* **26**, 281–286 (2020).

For peer review only - http://bmjopen.bmj.com/site/about/guidelines.xhtml

30. Tugwell, P. Economic evaluation of the management of pain in osteoarthritis. *Drugs* **52 Suppl 3**, 48–58 (1996).

31. Schnipper, L. E. *et al.* Updating the American Society of Clinical Oncology Value Framework: Revisions and Reflections in Response to Comments Received. *JCO* **34**, 2925–2934 (2016).

32. Adami, S. *et al.* Therapeutic value and innovation of a drug and criteria for evaluation: A multidisciplinary experience in the veneto region. *Global and Regional Health Technology Assessment* **2**, 107–113 (2015).

33. Capri, S. & Levaggi, R. Pricing policies in the pharmaceutical sector. *Drug Information Journal* **36**, 453–464 (2002).

34. Mejía, A., Gilardino, R., Kristensen, F. B. & Garrison, L. P. Value-Based Pricing in Latin America: How Far Away Are We? *Value in Health Regional Issues* **17**, 219–223 (2018).

35. Schey, C., Krabbe, P. F. M., Postma, M. J. & Connolly, M. P. Multi-criteria decision analysis (MCDA): Testing a proposed MCDA framework for orphan drugs. *Orphanet Journal of Rare Diseases* **12**, (2017).

36. Towse, A. & Barnsley, P. Approaches to identifying, measuring, and aggregating elements of value. *International journal of technology assessment in health care* **29**, 360–364 (2013).

37. Annemans, L. *et al.* Recommendations from the European Working Group for Value Assessment and Funding Processes in Rare Diseases (ORPH-VAL). *Orphanet journal of rare diseases* **12**, 50 (2017).

38. Danzon, P. M. Affordability Challenges to Value-Based Pricing: Mass Diseases, Orphan Diseases, and Cures. *Value in health : the journal of the International Society for Pharmacoeconomics and Outcomes Research* **21**, 252–257 (2018).

For peer review only

39. Skipka, G. *et al.* Methodological approach to determine minor, considerable, and major treatment effects in the early benefit assessment of new drugs. *Biometrical journal. Biometrische Zeitschrift* **58**, 43–58 (2016).

40. Linley, W. G. & Hughes, D. A. Societal views on NICE, cancer drugs fund and value-based pricing criteria for prioritising medicines: a cross-sectional survey of 4118 adults in Great Britain. *Health economics* **22**, 948–964 (2013).

41. Parmar, A., Jiao, T., Saluja, R. & Chan, K. K. W. Value-based pricing: Toward achieving a balance between individual and population gains in health benefits. *Cancer medicine* **9**, 94–103 (2020).

42. Augustovski, F. & McClellan, M. B. Current Policy and Practice for Value-Based Pricing. *Value in Health* **22**, S4–S6 (2019).

43. Picavet, E., Cassiman, D. & Simoens, S. Reimbursement of orphan drugs in Belgium: What (else) matters? *Orphanet Journal of Rare Diseases* **9**, (2014).

44. Nuijten, M. & Capri, S. Pricing of orphan drugs in oncology and rare diseases. *Journal of market access & health policy* **8**, 1838191 (2020).

45. Horgan, D. Treatment Is Worth a Lot - But Just How Much. *Biomedicine hub* **2**, 202–211 (2017).

46. Medic, G. *et al.* Do payers value rarity? An analysis of the relationship between disease rarity and orphan drug prices in Europe. *Journal of market access & health policy* **5**, 1299665 (2017).

47. Gutierrez, L., Patris, J., Hutchings, A. & Cowell, W. Principles for consistent value assessment and sustainable funding of orphan drugs in Europe. *Orphanet journal of rare diseases* **10**, 53 (2015).

48. Huskamp, H. A. Prices, profits, and innovation: examining criticisms of new psychotropic drugs' value. *Health affairs (Project Hope)* **25**, 635–646 (2006).

For peer review only

49. Sussex, J., Towse, A. & Devlin, N. Operationalizing value-based pricing of medicines : a taxonomy of approaches. *PharmacoEconomics* **31**, 1–10 (2013).

50. Denis, A. *et al.* Critical assessment of Belgian reimbursement dossiers of orphan drugs. *PharmacoEconomics* **29**, 883–893 (2011).

51. Persson, U. & Norlin, J. M. Multi-indication and Combination Pricing and Reimbursement of Pharmaceuticals: Opportunities for Improved Health Care through Faster Uptake of New Innovations. *Applied Health Economics and Health Policy* **16**, 157–165 (2018).

52. Takayama, A., Kobayashi, E., Nakamura, T. & Narukawa, M. Quantitative Assessment of Premium Rates for Clinical Usefulness in New Drug Price Calculation in Japan. *Therapeutic innovation & regulatory science* **51**, 582–588 (2017).

53. Barei, F. Value-added medicines: is there any room between market access challenges and 'patient centricity'? *Int. J. Pharm. Healthc. Mark.* **12**, 71–76 (2018).

54. Le Jeunne, C., Woronoff-Lemsi, M.-C., David, N. & de Sahb, R. La valeur ajoutée relative : quels outils pour l'évaluer ? *Therapie* **63**, 107–111 (2008).

55. Cañás, M., Buschiazzo, H. O. & Urtasun, M. A. Therapeutic value and price of the new pharmaceuticals commercialized in Argentina: Are they worth what they cost? *Salud colectiva* **15**, e1962 (2019).

56. Hansson, L. F., Norheim, O. F. & Ruyter, K. W. Equality, Explicitness, Severity, and Rigidity: The Oregon Plan Evaluated from a Scandinavian Perspective. *Journal of Medicine and Philosophy* **19**, 343–366 (1994).

57. Burkholder, R. Pricing and Value of Cancer Drugs. *JAMA oncology* **1**, 841–842 (2015).

58. McCabe, C., Claxton, K. & Tsuchiya, A. Orphan drugs and the NHS: should we value rarity? *BMJ* **331**, 1016–1019 (2005).

For peer review only

59. Philipson, T., Kamal-Bahl, S., Jena, A., Philipson, T. J. & Jena, A. B. Defining Value: The Need for a Longer, Broader View. *PharmacoEconomics* **35**, 669–672 (2017).

60. Walton, S. M., Basu, A., Mullahy, J., Hong, S. & Schumock, G. T. Measuring the Value of Pharmaceuticals in the US Health System. *PharmacoEconomics* **35**, 1–4 (2017).

61. Emanuel, E. J. *et al.* Drug Reimbursement Regulation in 6 Peer Countries. *JAMA Intern Med* **180**, 1510 (2020).

62. Vokinger, K. N. *et al.* Analysis of Launch and Postapproval Cancer Drug Pricing, Clinical Benefit, and Policy Implications in the US and Europe. *JAMA Oncol* **7**, e212026 (2021).

63. Moreau-Bachelard, C., Coquan, E. & Le Tourneau, C. Imputability of Adverse Events to Anticancer Drugs. *N Engl J Med* **380**, 1873–1874 (2019).

Figure 1. Flow diagram of search results

For peer review only

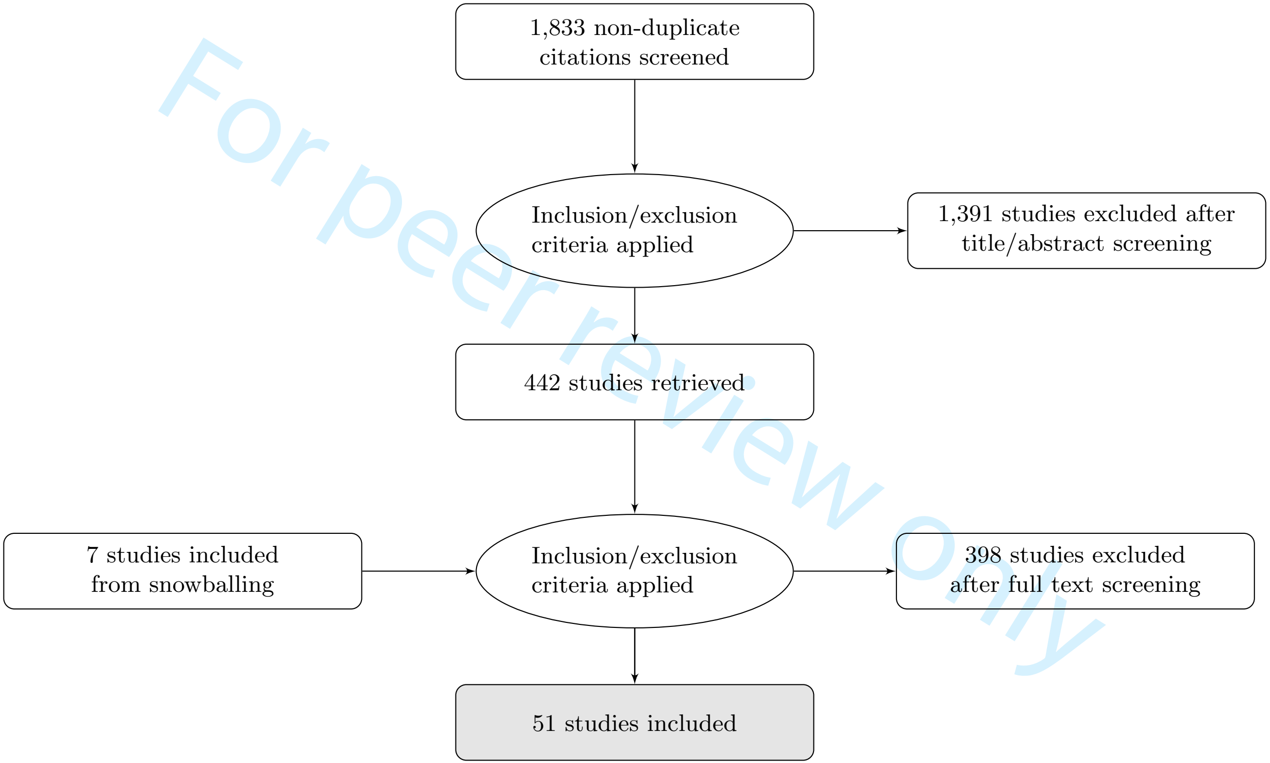

For peer review only

Appendix

Search Strategy
The search string contained (value or worth) or (“value based”) or (clinical* or medical* or therap* or cur* or pay* or drug* or pharmaceutical* or evaluat* or assess* or defin*). An additional operator was used so that there was a maximum of three words between the above terms and (valu* or worth). The second search string was (clinical* or medical* or therap* or cur* or patient*) and had an adjacent operator so that there was a maximum of three words benefit. The third search string was the following: ((drug* OR pharmac* OR medicine* OR medicat*) NEAR/20 pric*) OR AB= ((drug* OR pharmac* OR medicine* OR medicat*) NEAR/3 pric)).

Search Web of Science on the 18.12.2020

1. TI=((drug* OR pharmac* OR medicine* OR medicat*) NEAR/20 pric*) OR AB=((drug* OR pharmac* OR medicine* OR medicat*) NEAR/3 pric*)
   Indexes=SCI-EXPANDED, SSCI, A&HCI, CPCI-S, CPCI-SSH, BKCI-S, BKCI-SSH, ESCI,CCR-EXPANDED, IC Timespan=All years
2. TI=(value OR worth) OR TS=("value based") OR TS=((clinical* OR medical* OR therap* OR cur* OR pay* OR drug* OR pharmaceutical* OR evaluat* OR assess* OR defin*) NEAR/3 (valu* OR worth)) OR TS=((clinical* OR medical* OR therap* OR cur* OR patient*) NEAR/3 benefit)
   Indexes=SCI-EXPANDED, SSCI, A&HCI, CPCI-S, CPCI-SSH, BKCI-S, BKCI-SSH, ESCI, CCR-EXPANDED, IC Timespan=All years
3. #2 AND #1
   Indexes=SCI-EXPANDED, SSCI, A&HCI, CPCI-S, CPCI-SSH, BKCI-S, BKCI-SSH, ESCI,
   CCR-EXPANDED, IC Timespan=All years
4. #2 AND #1
   Refined by: LANGUAGES: (ENGLISH OR FRENCH OR GERMAN OR ITALIAN ORSPANISH)
   Indexes=SCI-EXPANDED, SSCI, A&HCI, CPCI-S, CPCI-SSH, BKCI-S, BKCI-SSH, ESCI, CCR-EXPANDED, IC Timespan=All year

Search Cochrane Library on the 18.12.2020

1. ((drug* OR pharmac* OR medicine* OR medicat*) NEAR/20 pric*):ti OR ((drug* OR pharmac* OR medicine* OR medicat*) NEAR/3 pric*):ab,kw
2. (value OR worth):ti OR 'value based':ti,ab,kw OR ((clinical* OR medical* OR therap* OR cur* OR pay* OR drug* OR pharmaceutical* OR evaluat* OR assess* OR defin*) NEAR/3 (valu* OR worth)):ti,ab,kw OR ((clinical* OR medical* OR therap* OR cur* OR patient*) NEAR/3 benefit):ti,ab,kw
3. #1 AND #2
4. Type a search term or use the S or MeSH buttons to compose

Search Scopus on the 18.12.2020

1. TITLE(value OR worth) OR TITLE-ABS-KEY("value based") OR TITLE-ABS-KEY((clinical* OR medical* OR therap* OR cur* OR pay* OR drug* OR pharmaceutical* OR evaluat* OR assess* OR defin*) W/3 (valu* OR worth)) OR TITLE-ABS-KEY((clinical* OR medical* OR therap* OR cur* OR patient*) W/3 benefit)
2. TITLE ((drug* OR pharmac* OR medicine* OR medicat*) W/20 pric*) OR ABS ((drug* OR pharmac* OR medicine* OR medicat*) W/3 pric*)) AND (TITLE (value OR worth) OR TITLE-ABS-KEY ("value based") OR TITLE-ABS-KEY ((clinical* OR medical* OR therap* OR cur* OR pay* OR drug* OR

For peer review only

pharmaceutical* OR evaluat* OR assess* OR defin*) W/3 (valu* OR worth)) OR TITLE-ABS-KEY ((clinical* OR medical* OR therap* OR cur* OR patient*) W/3 benefit)) AND (LIMIT-TO (LANGUAGE , "English") OR LIMIT-TO (LANGUAGE , "French") OR LIMIT-TO (LANGUAGE , "German") OR LIMIT-TO (LANGUAGE , "Spanish") OR LIMIT-TO (LANGUAGE , "Italian"))

3. (TITLE ((drug* OR pharmac* OR medicine* OR medicat*) W/20 pric*) OR ABS ((drug* OR pharmac* OR medicine* OR medicat*) W/3 pric*)) AND (TITLE (value OR worth) OR TITLE-ABS-KEY ("value based") OR TITLE-ABS-KEY ((clinical* OR medical* OR therap* OR cur* OR pay* OR drug* OR pharmaceutical* OR evaluat* OR assess* OR defin*) W/3 (valu* OR worth)) OR TITLE-ABS-KEY ((clinical* OR medical* OR therap* OR cur* OR patient*) W/3 benefit))
4. TITLE (value OR worth) OR TITLE-ABS-KEY ("value based") OR TITLE-ABS-KEY ((clinical* OR medical* OR therap* OR cur* OR pay* OR drug* OR pharmaceutical* OR evaluat* OR assess* OR defin*) W/3 (valu* OR worth)) OR TITLE-ABS-KEY ((clinical* OR medical* OR therap* OR cur* OR patient*) W/3
5. TITLE ((drug* OR pharmac* OR medicine* OR medicat*) W/20 pric*) OR ABS ((drug* OR pharmac* OR medicine* OR medicat*) W/3 pric*)

Search Regional Business News on the 18.12.2020

1. TI ((drug* OR pharmac* ORmedicine* OR medicat*) N20pric*) OR AB ((drug* OR pharmac* OR medicine* OR medicat*) N3 pric*)
2. TI (value OR worth) OR TI ("value based") OR TI ((clinical* OR medical* OR therap* OR cur* OR pay* OR drug* OR pharmaceutical* OR evaluat* OR assess* OR defin*) N3 (valu* OR worth)) OR TI ((clinical* OR medical* OR therap* OR cur* OR patient*) N3 benefit) OR AB ("value based") OR AB ((clinical* OR medical* OR therap* OR cur* OR pay* OR drug* OR pharmaceutical* OR evaluat* OR assess* OR defin*) N3 (valu* OR worth)) OR AB ((clinical* OR medical* ORtherap* OR cur* OR patient*) N3 benefit)
3. S1 AND S2

Search Medline on the 18.12.2020

1. (((MH "Drug Costs") OR (MH "Pharmaceutical Preparations+")) AND TX (pric*)) OR TI ((drug* OR pharmac* OR medicine* OR medicat*) N20 pric*) OR AB ((drug* OR pharmac* OR medicine* OR medicat*) N3 pric*
2. TI (value OR worth) OR TX ("value based") OR TX ((clinical* OR medical* OR therap* OR cur* OR pay* OR drug* OR pharmaceutical* OR evaluat* OR assess* OR defin*) N3 (valu* OR worth)) OR TX((clinical* OR medical* ORtherap* OR cur* OR patient*)N3 benefit)
3. S1 AND S2
4. S1 AND S2 with Limiters - Language:English, French, German, Italian, Spanish Expanders – Apply equivalent subjects Search modes - Find all mysearch term

Search Embase Session Results on the 18.12.2020

1. ('pharmacoeconomics'/exp OR 'drug'/exp) AND 'pric*':ti,ab,kw OR (((drug* OR pharmac* OR medicine* OR medicat*) NEAR/20 pric*):ti) OR (((drug* OR pharmac* OR medicine* OR medicat*) NEAR/3 pric*):ab,kw)
2. value:ti OR worth:ti OR 'value based':ti,ab,kw OR (((clinical* OR medical* OR therap* OR cur* OR pay* ORdrug* OR pharmaceutical* OR evaluat* OR assess* OR defin*)

For peer review only

NEAR/3 (valu* OR worth)):ti,ab,kw) OR (((clinical* OR medical* OR therap* OR cur* OR patient*) NEAR/3 benefit):ti,ab,kw)

3. #1 AND #2
4. #1 AND #2 AND [conference abstract]/lim
5. #1 AND #2 NOT [conference abstract]/lim
6. #1 AND #2 NOT [conference abstract]/lim AND ([english]/lim OR [french]/lim OR [german]/lim OR [italian]/lim OR [spanish]/lim)

EconLit on the 18.12.2020

1. TI ((drug* OR pharmac* OR medicine* OR medicat*) N20 pric*) OR AB ((drug* OR pharmac* OR medicine* OR medicat*) N3 pric*)
2. TI (value OR worth) OR TI ("value based") OR TI ((clinical* OR medical* OR therap* OR cur* OR pay* OR drug* OR pharmaceutical* OR evaluat* OR assess* OR defin*) N3 (valu* OR worth)) OR TI ((clinical* OR medical* OR therap* OR cur* OR patient*) N3 benefit) OR AB ("value based") OR AB ((clinical* OR medical* OR therap* OR cur* OR pay* OR drug* OR pharmaceutical* OR evaluat* OR assess* OR defin*) N3 (valu* OR worth)) OR AB ((clinical* OR medical* OR therap* OR cur* OR patient*) N3 benefit)
3. S1 AND S2

CINAHL on the 18.12.2020

1. ((MH "Drugs+") AND (TI (pric*) OR AB (pric*))) OR TI ((drug* OR pharmac* OR medicine* OR medicat*) N20 pric*) OR AB ((drug* OR pharmac* OR medicine* OR medicat*) N3 pric*)
2. TI (value OR worth) OR TI ("value based") OR TI ((clinical* OR medical* OR therap* OR cur* OR pay* OR drug* OR pharmaceutical* OR evaluat* OR assess* OR defin*) N3 (valu* OR worth)) OR TI ((clinical* OR medical* OR therap* OR cur* OR patient*) N3 benefit) OR AB ("value based") OR AB ((clinical* OR medical* OR therap* OR cur* OR pay* OR drug* ORpharmaceutical* OR evaluat*OR assess* OR defin*) N3(valu* OR worth)) OR AB ((clinical* OR medical* OR therap* OR cur* OR patient*)N3 benefit)
3. S1 AND S2
4. S1 AND S2

Search on Business Source Premier on 18.12.2020

1. DE "DRUG prices" OR DE "PHARMACEUTICAL reference pricing" OR TI ((drug* OR pharmac* OR medicine* OR medicat*) N20 pric*) OR AB ((drug* OR pharmac* OR medicine* OR medicat*) N3 pric*)
2. TI (value OR worth) OR TI ("value based") OR TI ((clinical* OR medical* OR therap* OR cur* OR pay* OR drug* OR pharmaceutical* OR evaluat* OR assess* OR defin*) N3 (valu* OR worth)) OR TI ((clinical* OR medical* OR therap* OR cur* OR patient*) N3 benefit) OR AB ("value based") OR AB ((clinical* OR medical* OR therap* OR cur* OR pay* OR drug* OR pharmaceutical* OR evaluat* OR assess* OR defin*) N3 (valu* OR worth)) OR AB ((clinical* OR medical* OR therap* OR cur* OR patient*) N3 benefit)
3. S1 AND S2

For peer review only

Preferred Reporting Items for Systematic reviews and Meta-Analyses extension for Scoping Reviews (PRISMA-ScR) Checklist

SECTION	ITEM	PRISMA-ScR CHECKLIST ITEM	REPORTED ON PAGE #
**TITLE**			
Title	1	Identify the report as a scoping review.	1, 2, 3
**ABSTRACT**			
Structured summary	2	Provide a structured summary that includes (as applicable): background, objectives, eligibility criteria, sources of evidence, charting methods, results, and conclusions that relate to the review questions and objectives.	2
**INTRODUCTION**			
Rationale	3	Describe the rationale for the review in the context of what is already known. Explain why the review questions/objectives lend themselves to a scoping review approach.	3
Objectives	4	Provide an explicit statement of the questions and objectives being addressed with reference to their key elements (e.g., population or participants, concepts, and context) or other relevant key elements used to conceptualize the review questions and/or objectives.	3
**METHODS**			
Protocol and registration	5	Indicate whether a review protocol exists; state if and where it can be accessed (e.g., a Web address); and if available, provide registration information, including the registration number.	Registration information on Prospero number CRD42021268174.
Eligibility criteria	6	Specify characteristics of the sources of evidence used as eligibility criteria (e.g., years considered, language, and publication status), and provide a rationale.	4
Information sources*	7	Describe all information sources in the search (e.g., databases with dates of coverage and contact with authors to identify additional sources), as well as the date the most recent search was executed.	3, 4
Search	8	Present the full electronic search strategy for at least 1 database, including any limits used, such that it could be repeated.	3,4
Selection of sources of evidence†	9	State the process for selecting sources of evidence (i.e., screening and eligibility) included in the scoping review.	3, 4
Data charting process‡	10	Describe the methods of charting data from the included sources of evidence (e.g., calibrated forms or forms that have been tested by the team before their use, and whether data charting was done independently or in duplicate) and any processes for obtaining and confirming data from investigators.	3,4

For peer review only - http://bmjopen.bmj.com/site/about/guidelines.xhtml

SECTION	ITEM	PRISMA-ScR CHECKLIST ITEM	REPORTED ON PAGE #
Data items	11	List and define all variables for which data were sought and any assumptions and simplifications made.	4
Critical appraisal of individual sources of evidence§	12	If done, provide a rationale for conducting a critical appraisal of included sources of evidence; describe the methods used and how this information was used in any data synthesis (if appropriate).	NA
Synthesis of results	13	Describe the methods of handling and summarizing the data that were charted.	4
**RESULTS**			
Selection of sources of evidence	14	Give numbers of sources of evidence screened, assessed for eligibility, and included in the review, with reasons for exclusions at each stage, ideally using a flow diagram.	Figure 1, page 11
Characteristics of sources of evidence	15	For each source of evidence, present characteristics for which data were charted and provide the citations.	Tables 2-4
Critical appraisal within sources of evidence	16	If done, present data on critical appraisal of included sources of evidence (see item 12).	NA
Results of individual sources of evidence	17	For each included source of evidence, present the relevant data that were charted that relate to the review questions and objectives.	Tables 2-4
Synthesis of results	18	Summarize and/or present the charting results as they relate to the review questions and objectives.	5
**DISCUSSION**			
Summary of evidence	19	Summarize the main results (including an overview of concepts, themes, and types of evidence available), link to the review questions and objectives, and consider the relevance to key groups.	8
Limitations	20	Discuss the limitations of the scoping review process.	9
Conclusions	21	Provide a general interpretation of the results with respect to the review questions and objectives, as well as potential implications and/or next steps.	8, 9, 10
**FUNDING**			
Funding	22	Describe sources of funding for the included sources of evidence, as well as sources of funding for the scoping review. Describe the role of the funders of the scoping review.	1

JBI = Joanna Briggs Institute; PRISMA-ScR = Preferred Reporting Items for Systematic reviews and Meta-Analyses extension for Scoping Reviews.
* Where *sources of evidence* (see second footnote) are compiled from, such as bibliographic databases, social media platforms, and Web sites.
† A more inclusive/heterogeneous term used to account for the different types of evidence or data sources (e.g., quantitative and/or qualitative research, expert opinion, and policy documents) that may be eligible in a scoping review as opposed to only studies. This is not to be confused with *information sources* (see first footnote).

For peer review only - http://bmjopen.bmj.com/site/about/guidelines.xhtml

‡ The frameworks by Arksey and O'Malley (6) and Levac and colleagues (7) and the JBI guidance (4, 5) refer to the process of data extraction in a scoping review as data charting.
§ The process of systematically examining research evidence to assess its validity, results, and relevance before using it to inform a decision. This term is used for items 12 and 19 instead of "risk of bias" (which is more applicable to systematic reviews of interventions) to include and acknowledge the various sources of evidence that may be used in a scoping review (e.g., quantitative and/or qualitative research, expert opinion, and policy document).

From: Tricco AC, Lillie E, Zarin W, O'Brien KK, Colquhoun H, Levac D, et al. PRISMA Extension for Scoping Reviews (PRISMAScR): Checklist and Explanation. Ann Intern Med. 2018;169:467–473. doi: 10.7326/M18-0850.

For peer review only
